# c-di-GMP inhibits LonA-dependent proteolysis of TfoY in *Vibrio cholerae*

**DOI:** 10.1371/journal.pgen.1008897

**Published:** 2020-06-26

**Authors:** Avatar Joshi, Samar A. Mahmoud, Soo-Kyoung Kim, Justyne L. Ogdahl, Vincent T. Lee, Peter Chien, Fitnat H. Yildiz

**Affiliations:** 1 Department of Microbiology and Environmental Toxicology, University of California Santa Cruz, Santa Cruz, California, United States of America; 2 Department of Biochemistry and Molecular Biology, Molecular and Cellular Biology Graduate Program, University of Massachusetts, Amherst, Massachusetts, United States of America; 3 Department of Cell Biology and Molecular Genetics, University of Maryland, College Park, Maryland, United States of America; Michigan State University, UNITED STATES

## Abstract

The LonA (or Lon) protease is a central post-translational regulator in diverse bacterial species. In *Vibrio cholerae*, LonA regulates a broad range of behaviors including cell division, biofilm formation, flagellar motility, c-di-GMP levels, the type VI secretion system (T6SS), virulence gene expression, and host colonization. Despite LonA’s role in cellular processes critical for *V*. *cholerae’s* aquatic and infectious life cycles, relatively few LonA substrates have been identified. LonA protease substrates were therefore identified through comparison of the proteomes of wild-type and Δ*lonA* strains following translational inhibition. The most significantly enriched LonA-dependent protein was TfoY, a known regulator of motility and the T6SS in *V*. *cholerae*. Experiments showed that TfoY was required for LonA-mediated repression of motility and T6SS-dependent killing. In addition, TfoY was stabilized under high c-di-GMP conditions and biochemical analysis determined direct binding of c-di-GMP to LonA results in inhibition of its protease activity. The work presented here adds to the list of LonA substrates, identifies LonA as a c-di-GMP receptor, demonstrates that c-di-GMP regulates LonA activity and TfoY protein stability, and helps elucidate the mechanisms by which LonA controls important *V*. *cholerae* behaviors.

## Author summary

This study provides insights into the mechanisms and consequences of LonA-mediated regulated proteolysis in *Vibrio cholerae*, the causal organism of the acute diarrheal disease cholera that is endemic in more than 47 countries across the globe. Lon is broadly conserved in bacterial systems; uncovering the molecular connection between c-di-GMP signaling and LonA-mediated proteolysis of *V*. *cholerae* will provide conceptual frameworks for the development of intervention strategies to combat virulence by bacterial pathogens.

## Introduction

Regulated proteolysis is a critical cellular mechanism that helps cells maintain homeostasis and regulate diverse processes [1–3]. The LonA (or Lon) protease is present across all domains of life and plays a central role in maintaining cellular homeostasis. LonA facilitates the turnover of misfolded, damaged, or unused proteins, a process which frees amino acids for use in other cellular machinery [1–3]. LonA’s central role in governing cellular behaviors is highlighted by the gross dysregulation of wide-ranging cellular processes in its absence. For example, in many bacterial species, loss of *lonA* results in aberrant cell division, susceptibility to stressors such as UV irradiation and heat shock, as well as aberrant control of motility, biofilm formation, quorum sensing, virulence factor production, and host colonization. Indeed, deletion of *lonA* significantly reduces the *in vivo* fitness of every pathogenic bacteria in which it has been tested [4–12].

LonA belongs to the superfamily of ATPases associated with diverse cellular activities (AAA+ ATPases) [1–3]. LonA monomers assemble into a barrel shaped hexamer, which utilizes successive rounds of ATP-hydrolysis to bind, unwind, and translocate proteins into a central chamber where catalytic serine and lysine residues irreversibly proteolyze substrates [1–3,13,14]. Because proteolysis is irreversible, the selectivity of AAA+ proteases must be carefully controlled [1–3,14,15]. In some cases, AAA+ proteases recognize their substrates directly, via recognition of conserved motifs known as degrons [1–3,13,14]. In other cases, additional specificity factors, known as adaptors, or other small signaling molecules can modulate the rate of proteolysis. To date, only two LonA adaptor proteins have been identified [16,17]. The first LonA adaptor, known as SmiA, was identified in *Bacillus subtilis* and coordinates proteolysis of the swarming motility master regulator SwrA [16]. The second adaptor, known as HspQ, is a specificity-enhancing factor that allosterically activates *Yersinia pestis* Lon protease against diverse substrates including a small histone-like protein YmoA, which controls activation of the type III secretion system [17]. LonA has also been shown to respond to diverse signals *in vitro*, such as polyphosphate, cyclic AMP, guanosine tetraphosphate, c-di-GMP, and DNA; however, relatively little is known regarding the physiological consequences of these molecules *in vivo* [18–20].

LonA plays a critical role in the infection cycle of *Vibrio cholerae*, the facultative human pathogen responsible for the acute diarrheal disease cholera. *V*. *cholerae* remains a threat to global public health. There are estimated to be 1.3–4.0 million cases of cholera and 21,000–143,000 deaths worldwide each year [21]. We previously demonstrated LonA’s importance in *V*. *cholerae* pathogenesis as deletion of *lonA* results in a severe colonization defect in the infant mouse model [4]. LonA positively regulates biofilm formation but negatively regulates motility, the toxin co-regulated pilus and cholera toxin [4,8]. In addition, LonA negatively regulates the type VI secretion system (T6SS), a contact dependent contractile spear that translocates toxins into neighboring prokaryotic and eukaryotic cells [4,22]. LonA also functions as an activator and a repressor of c-di-GMP pools in planktonic and biofilm grown cells, respectively [4]. Finally, deletion of *lonA* results in filamentation of cells, suggesting it plays a role in cell septation [4]. Collectively, these phenotypes demonstrate the significance of LonA regulated cellular processes in *V*. *cholerae* pathogenesis and environmental survival. To date, only two known LonA substrates have been identified in *V*. *cholerae*. The first is FliA, an alternative sigma factor (σ^28^) that coordinates the activation of late stage flagellar genes and the repression of virulence gene expression [8]. The second is the quorum sensing master regulator HapR, which is proteolyzed by LonA upon heat shock in order to induce biofilm formation [23]. LonA proteolysis of FliA or HapR is highly condition dependent and is not sufficient to explain a majority of the phenotypes observed in a *lonA* mutant.

In the current study, using a quantitative proteomics approach, we identify the T6SS and motility regulator TfoY as a LonA target. We show that the hyper activation of motility and T6SS-dependent killing in the Δ*lonA* strain are due to the absence of LonA-mediated degradation of TfoY. Further, we find that c-di-GMP represses LonA proteolysis of TfoY *in vivo* and show that c-di-GMP directly binds to LonA and inhibits its activity *in vitro*. Finally, we demonstrate the significance of LonA and TfoY mediated regulation of motility and T6SS-dependent killing phenotypes in strains with high and low cellular levels of c-di-GMP relative to WT. Our work provides the first *in vivo* evidence that LonA is a true c-di-GMP receptor protein and suggests how this second messenger can temper the levels of TfoY through changes in regulated degradation.

## Results

### Whole proteome analysis identifies TfoY as a putative LonA substrate

**Fig 1:**
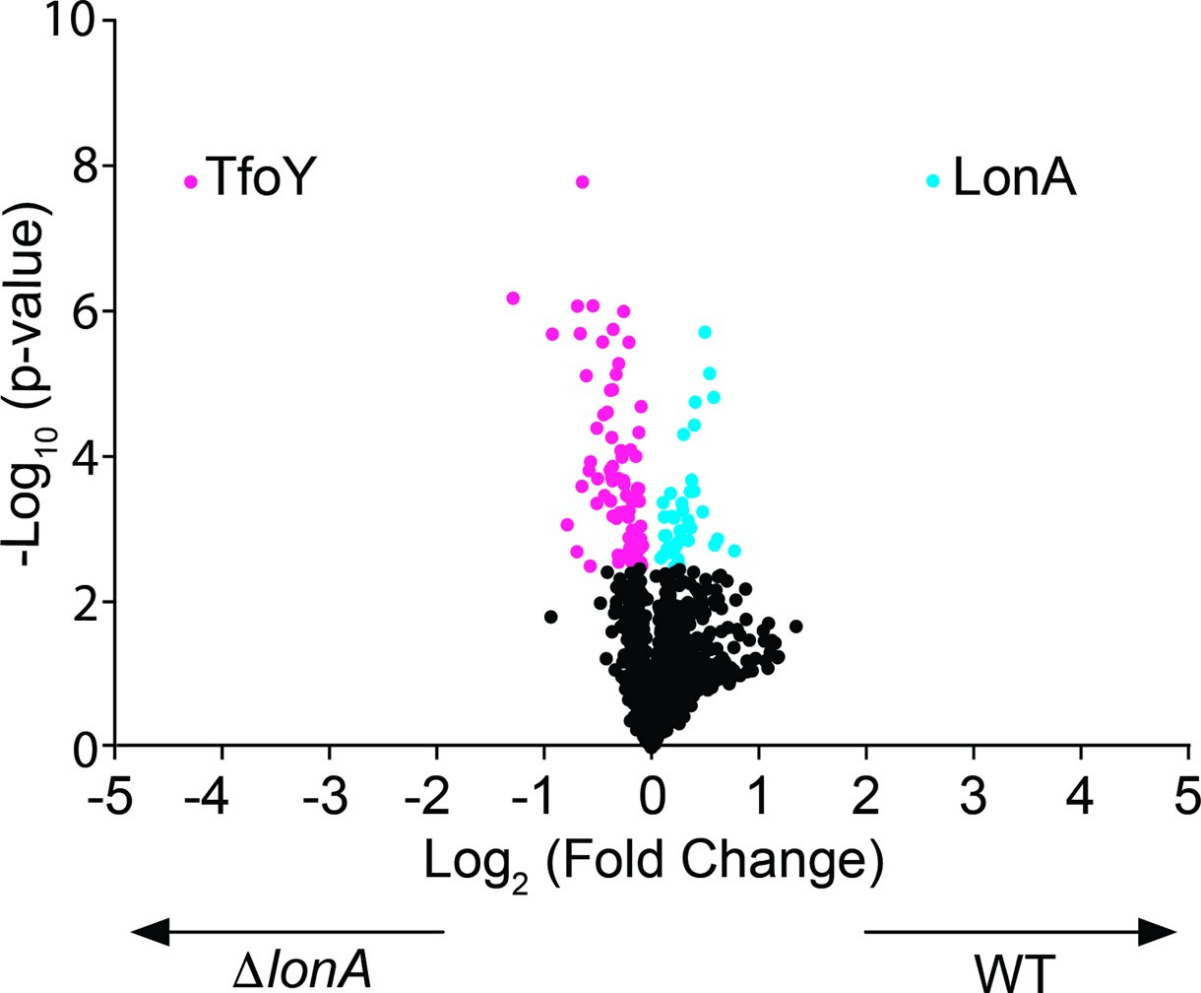
TfoY is significantly enriched in the Δ*lonA* mutant relative to wild-type. A volcano plot of proteins enriched in WT and Δ*lonA* mutant after translational inhibition. The proteomes of WT and Δ*lonA* strains (n = 5) that had been grown to an OD_600_ = 1.0 and exposed to the translational inhibitor chloramphenicol for 1-hour were analyzed by TMT-labeling and LC-MS/MS. A student’s t-test using a Benjamini-Hochberg FDR cutoff of 5% was used to identify proteins that were statistically significantly enriched. Proteins enriched in Δ*lonA* relative to WT are shown in pink. Proteins enriched in WT relative to Δ*lonA* are shown in blue.

The substrates controlled by LonA in *V*. *cholerae* remain poorly characterized. Since the stability of a protein targeted for degradation is directly dependent upon the protease or proteases that degrade it, we sought to perform a global analysis of the relative enrichment of proteins in wild-type (WT) and Δ*lonA* strains. To identify proteins whose stability are dependent upon LonA, we used tandem mass tag (TMT)-labeling coupled with liquid chromatography tandem mass spectrometry (LC-MS/MS) to quantify the proteomes of WT and a Δ*lonA* mutant one hour after treatment with the translational inhibitor chloramphenicol. We reasoned that these conditions would reveal the most striking differences for LonA substrates (Fig 1). We identified 80 proteins to be significantly enriched in the Δ*lonA* strain (S1 Table), suggesting that these proteins are either proteolyzed by LonA or are regulated in a LonA-dependent pathway. In addition, we identified 38 proteins to be significantly enriched in WT relative to the Δ*lonA* strain (S2 Table), suggesting that LonA positively impacts production of these proteins through indirect means.

The proteins identified in our analysis are predicted to be involved in a wide array of metabolic activities such as amino acid and protein biosynthesis, central intermediary metabolism, energy metabolism, fatty acid and phospholipid biosynthesis, the synthesis of nucleosides and nucleotides, and DNA metabolism. In addition, there was significant enrichment of hypothetical proteins and those involved in protein fate, secretion, or predicted to have regulatory functions. The most significantly enriched protein identified in our analysis was the transcriptional regulator TfoY (4.28 log_2_ fold increase), which was recently shown to lead to significant increases in motility and the T6SS, two behaviors that LonA represses [24]. Given that TfoY was the most abundant protein identified in our analysis and LonA control over TfoY could explain multiple Δ*lonA* phenotypes, we chose to focus our analysis on TfoY.

### TfoY stability is controlled by LonA

**Fig 2:**
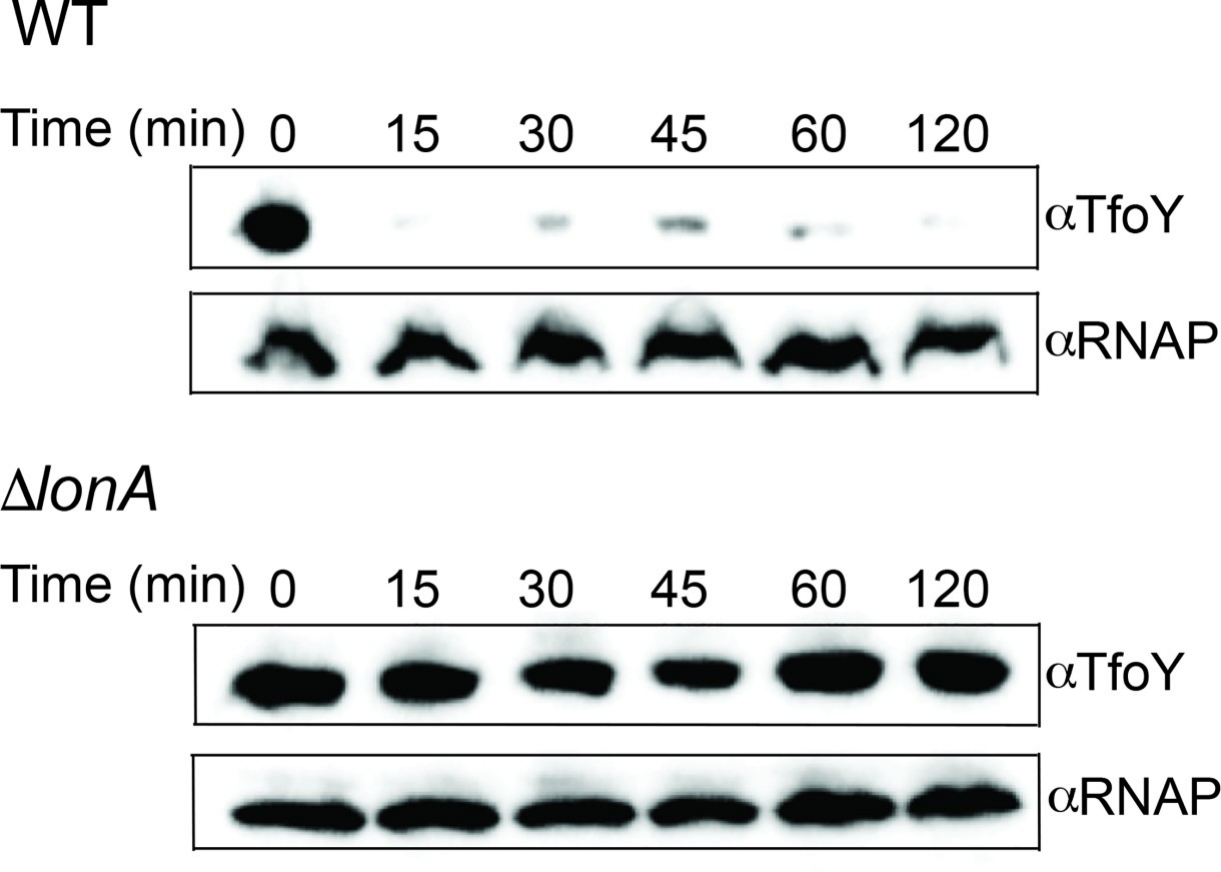
TfoY stability depends on the LonA protease. *In vivo* stability of TfoY after translational inhibition. TfoY was overproduced from the Tn7 locus in WT and a Δ*lonA* strain. Stability of TfoY was analyzed by western blot using an antibody against TfoY 1-hour post translational inhibition via chloramphenicol. RNAP was used as a biomass loading control.

To validate that TfoY stability is dependent upon LonA, we performed an *in vivo* protein stability assay. Since the conditions that lead to TfoY production remain to be fully elucidated, we placed the *tfoY* gene under the control of the P*tac* promoter at the Tn7 locus on the chromosome in both WT and Δ*lonA* strains [25]. We then induced TfoY production via the addition of IPTG and tested TfoY stability as a function of time after translational inhibition. We observed that TfoY is highly unstable in the WT genetic background, with the majority of TfoY protein degraded within 15 minutes (Fig 2A). In contrast, TfoY was stabilized in the absence of LonA (Fig 2B). Indeed, we observed little to no signs of degradation for at least two hours, suggesting that LonA is the major factor governing TfoY protein stability in *V*. *cholerae*.

### LonA represses motility and the type VI secretion system via TfoY

**Fig 3:**
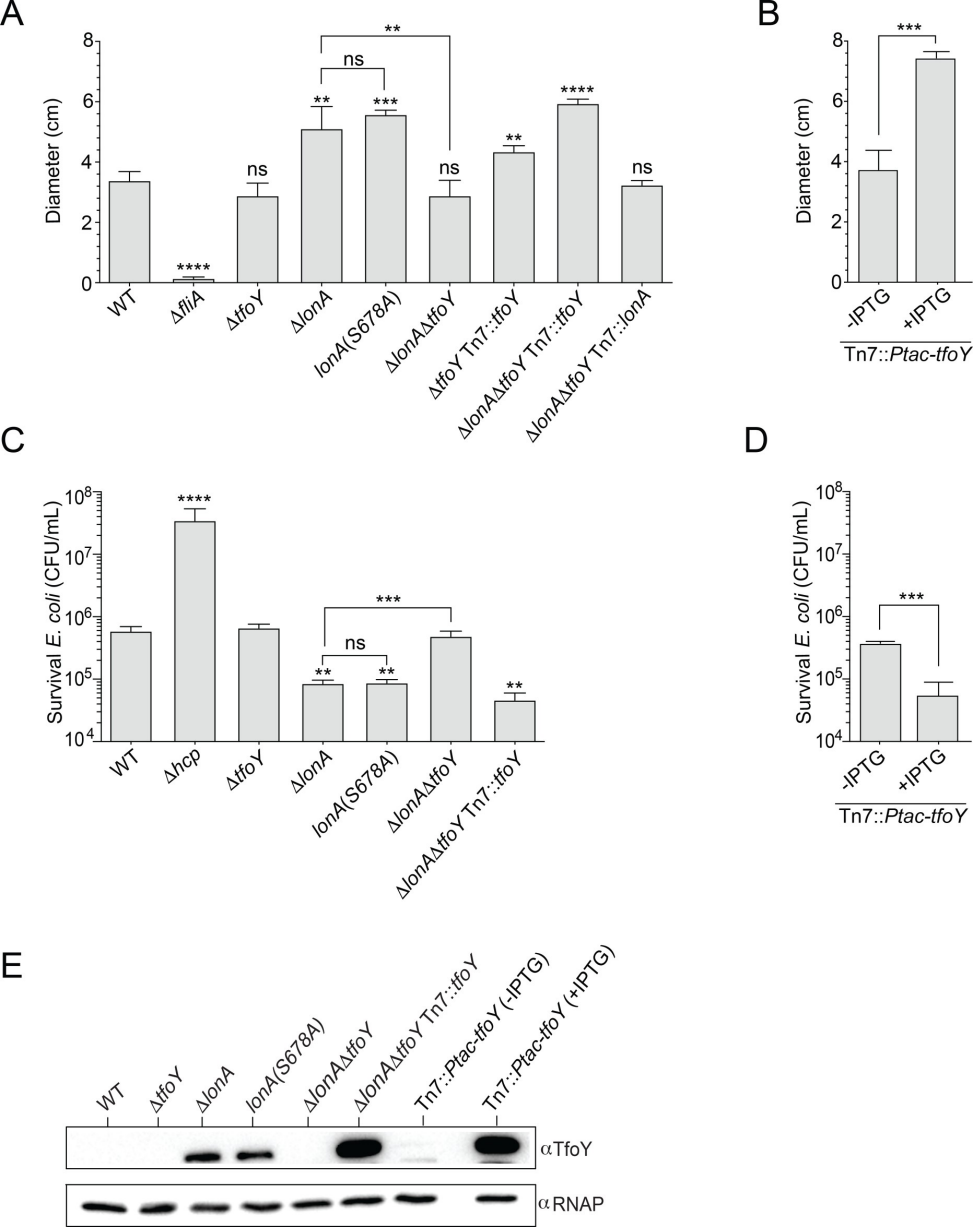
LonA represses motility and the T6SS through TfoY. Quantification of flagellar motility and T6SS killing experiments. For motility assays, single colonies were stabbed into LB soft agar plates (0.3% agar) and incubated at 30°C for approximately 18 hours. (A) Swimming motility phenotypes of WT, Δ*fliA* (negative control) and various Δ*lonA* and/or Δ*tfoY* deletions as well as their complementation strains from the Tn7 site. (B) Overexpression of *tfoY* from the *Ptac* promoter in plates with and without IPTG. (C) The T6SS killing phenotypes of various *tfoY* and *lonA* deletion mutants as well as their complementation strains were analyzed. T6SS killing was determined by enumerating the survival of *E*. *coli* strain MC4100, which is susceptible to T6SS attack. In addition, *hcp* was included as a negative control for T6SS dependent killing and *lonA*(S678A) as a control for LonA-dependent proteolysis. (D) Overexpression of *tfoY* from the *Ptac* promoter on plates with or without IPTG. Motility and T6SS-dependent killing experiments represent the average and SD of at least three independent experiments. Statistical analysis was performed using an unpaired Student’s t-test. Statistical values indicated are (**p<0.01, ***p < .001, and ****p < .0001). (E) Abundance of natively produced TfoY as well as overexpressed TfoY from the *Ptac* promoter.

LonA is a repressor of motility and the T6SS, while TfoY enhances both pathways [4,24]. Given that TfoY stability is dependent upon LonA, one explanation for the hypermotility and increased T6SS-dependent killing observed in the Δ*lonA* background is the increased abundance and stability of TfoY protein. To determine if dysregulation of TfoY could account for the increased activation of motility and T6SS-dependent killing observed in the Δ*lonA* strain, we assessed the motility and T6SS-dependent killing phenotypes of WT, Δ*tfoY*, Δ*lonA*, and Δ*lonA*Δ*tfoY* as well as in a *tfoY* overproducing strain (Fig 3A–3D). The lack of *tfoY* did not result in statistically significant differences in motility or T6SS mediated killing relative to WT, suggesting that TfoY production is tightly regulated under these conditions. Consistent with our previous work, the Δ*lonA* strain exhibited increased motility and T6SS killing [4]. Furthermore, strains harboring a mutation in the active site of LonA, where the catalytic serine is replaced with an alanine (LonA^S678A^), exhibited motility and T6SS-dependent killing phenotypes similar to a Δ*lonA* strain, indicating that LonA’s proteolytic activity is necessary for repression of motility and T6SS-dependent killing. Consistent with our hypothesis, we observed that deletion of *tfoY* in the Δ*lonA* strain restored motility and T6SS-dependent killing to WT levels. We also observed that overproduction of TfoY leads to enhanced motility and T6SS-dependent killing, which is consistent with TfoY’s known role as an activator of the T6SS and motility (Fig 3B and 3D) [24,26]. In addition, we assessed levels of TfoY in *lonA* and *tfoY* mutant strains relative to WT (Fig 3E). We observed that detection of TfoY is dependent upon the presence of a functional LonA and that complementation of *tfoY* in the Δ*lonA*Δ*tfoY* mutant restored detection of TfoY. The high levels of TfoY in the complemented strain, which harbors 500 base pairs of the upstream regulatory sequence, suggests that additional regulatory factors may be present at the native locus of *tfoY*. Finally, overexpression of *tfoY* from the *Ptac* promoter leads to large increases in TfoY. Taken together, these findings suggest that LonA tempers motility and T6SS-dependent killing in *V*. *cholerae* by controlling cellular levels of TfoY.

### LonA controls multiple cellular processes independently of TfoY

**Fig 4:**
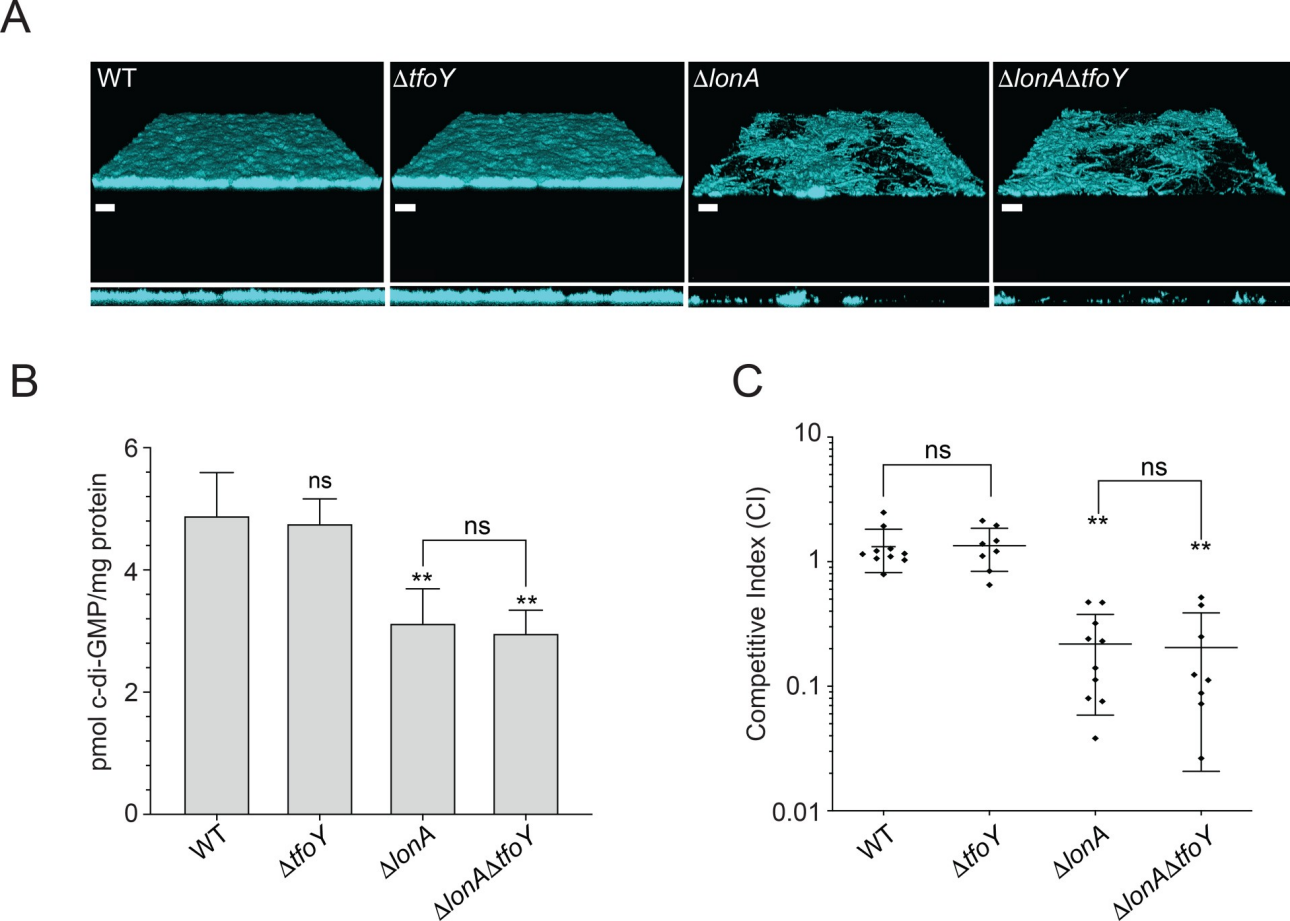
LonA regulates biofilm formation, cellular c-di-GMP levels, and intestinal colonization through TfoY independent mechanisms. Analysis of biofilm formation by CLSM, cellular c-di-GMP levels by LC-MS/MS, and intestinal colonization by *in vivo* competition assays. (A) Top-down and orthogonal views of mature biofilms formed by WT, Δ*tfoY*, Δ*lonA*, Δ*lonA*Δ*tfoY* mutants that contained *gfp* at the Tn7 locus. Scale bars are 40μm. (B) Cellular levels of c-di-GMP in WT, Δ*tfoY*, Δ*lonA*, Δ*lonA*Δ*tfoY* that had been grown to exponential phase (OD_600_ = 0.4) and analyzed for global c-di-GMP by LC-MS/MS. (C) Competitive index of *V*. *cholerae* strains. Otherwise WT strain (Δ*lacZ*) was co-inoculated with the strains indicated at a 1:1 ratio into 5-day old infant mice. The number of bacteria per intestine was determined 20 to 22 h post inoculation. The competitive index (CI) was determined as the output ratio of mutant to WT cells divided by the input ratio of mutant to WT cells per gram of intestine. Statistical analysis for panel B used a One-way ANOVA with Tukey’s post-hoc analysis. Statistical analysis for panel C used Wilcoxon’s signed rank test (*p<0.05, **p<0.01).

In addition to regulating motility and the T6SS, LonA is also responsible for modulating cellular processes that contribute to biofilm formation, intracellular pools of c-di-GMP, and intestinal colonization [4]. We wondered what role, if any, TfoY may play in these Δ*lonA* phenotypes. We evaluated the impact of TfoY on *lonA* biofilm formation using fluorescently labeled *V*. *cholerae* WT, Δ*tfoY*, Δ*lonA*, and Δ*lonA*Δ*tfoY* strains and confocal laser scanning microscopy (CLSM) (Fig 4A). We found that the biofilm-forming ability of the Δ*tfoY* strain is not altered, and that Δ*lonA* and Δ*lonA*Δ*tfoY* strains formed biofilms with similar properties (S3 Table) suggesting that LonA regulation of *tfoY* is not responsible for the aberrant biofilm formation observed in Δ*lonA*. In addition, TfoY appears to be dispensable for biofilm formation under the conditions used in this study.

Deletion of *lonA* also results in decreased levels of global c-di-GMP during exponential growth [4]. Intracellular pools of c-di-GMP are elevated through enzymes known as diguanylate cyclases (DGC) and decreased by phosphodiesterases (PDE). Thus, we wondered whether in the absence of *lonA*, TfoY may accumulate and lead to lower levels of c-di-GMP. We used LC-MS/MS to quantify global pools of c-di-GMP in WT, Δ*tfoY*, Δ*lonA*, and Δ*lonA*Δ*tfoY* strains (Fig 4B). Consistent with our previous analysis, we found that strains lacking *lonA* had lower levels of c-di-GMP [4]. However, we did not observe statistically significant differences between WT and Δ*tfoY* or between Δ*lonA* and Δ*lonA*Δ*tfoY* strains. This suggests that LonA’s control over c-di-GMP levels under these conditions occurs through a TfoY-independent pathway.

Finally, to determine what role the LonA and TfoY regulatory circuit might play during intestinal colonization, we performed an *in vivo* competition experiment by competing WT, Δ*tfoY*, Δ*lonA*, and Δ*lonA*Δ*tfoY* strains against a *lacZ-* WT strain in the infant mouse intestinal colonization model (Fig 4C). We did not observe a statistically significant difference between the Δ*lonA*Δ*tfoY* strain relative to the Δ*lonA* strain. In addition, Δ*tfoY* did not exhibit any competition defect relative to WT. We conclude that TfoY is not a significant factor involved during intestinal colonization of the infant mouse in either WT or Δ*lonA* genetic backgrounds.

### LonA activity and TfoY stability are modulated by c-di-GMP *in vivo*

**Fig 5:**
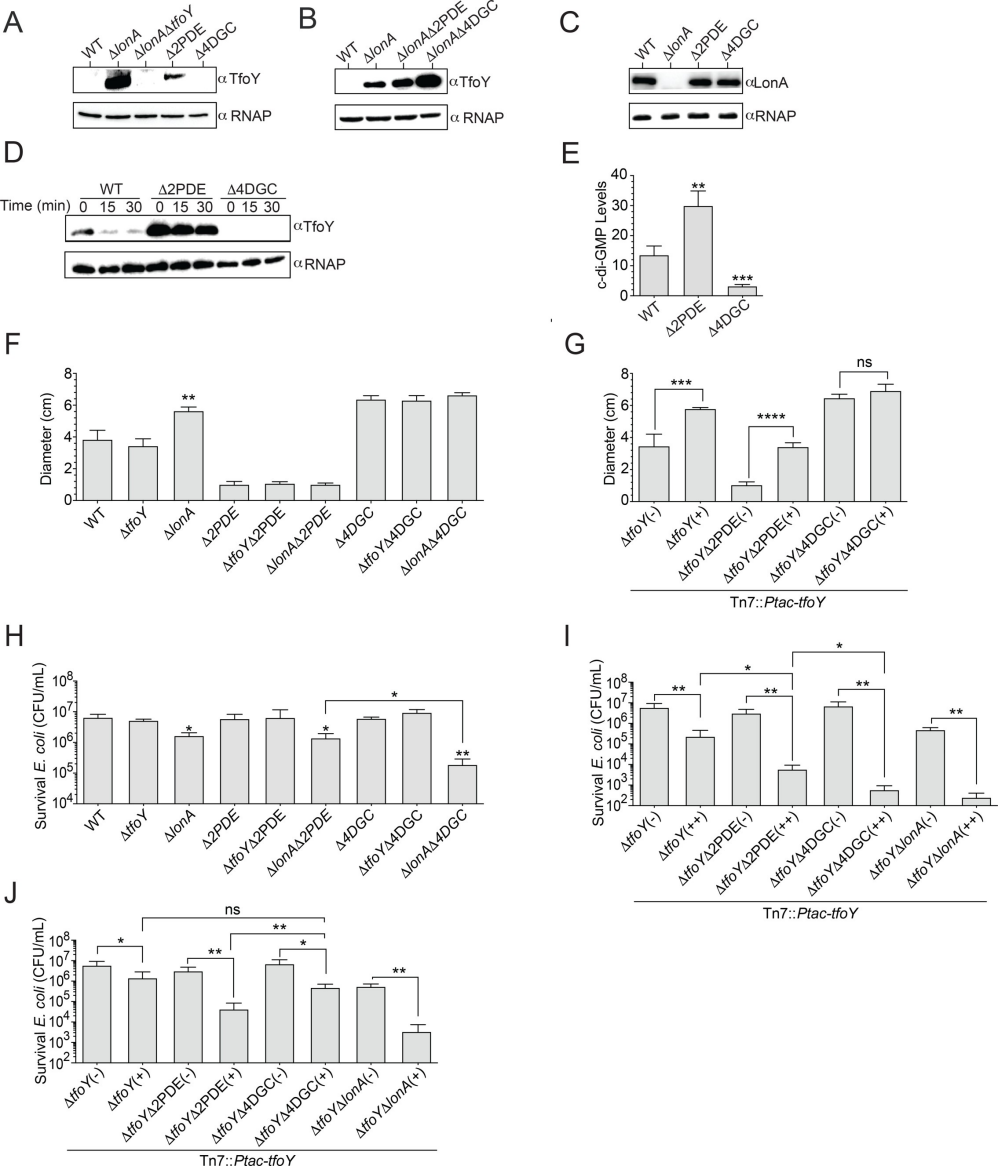
TfoY stability is influenced by c-di-GMP. *In vivo* abundance and stability of TfoY in high and low c-di-GMP genetic backgrounds relative to WT. (A) Abundance of natively produced TfoY in WT, Δ*lonA*, a mutant lacking two phosphodiesterases (Δ2PDE; Δ*rocS*Δ*cdgJ*) as well as a strain lacking four diguanylate cyclases (Δ4DGC; Δ*cdgD*Δ*cdgH*Δ*cdgK*Δ*cdgL*). (B) Abundance of TfoY in WT, Δ*lonA*, Δ*lonA*Δ2PDE, Δ*lonA*Δ4DGC strains. (C) Abundance of LonA in WT, Δ2PDE, and Δ4DGC strains. (D) TfoY was overproduced from the Tn7 locus in WT, Δ2PDE and Δ4DGC strains. Overproduction of TfoY was achieved via the addition of 0.1mM IPTG for 2 hours. Levels of TfoY were assessed immediately before and after translational inhibition via chloramphenicol. (E) Prior to translational inhibition, 40mLs of culture was spun down and analyzed for global c-di-GMP by LC-MS/MS. A One-way ANOVA using Dunnet’s multiple comparisons test was used for statistical analysis (**p<0.01, ***p < .001). Abundance and stability of TfoY was analyzed by western blot using an αTfoY antibody. RNAP was used as a control for sample loading in all western blots. Levels of LonA were analyzed by western blot using a αLonA antibody. (F) Swimming motility phenotypes of Δ*tfoY* and Δ*lonA* deletions in WT, Δ2PDE, and Δ4DGC strains. (G) Overexpression of *tfoY* from the *Ptac* promoter in plates with (+) and without (-) IPTG. For motility assays, single colonies were stabbed into LB soft agar plates (0.3% agar) and incubated at 30°C for approximately 18 hours. (H) The T6SS killing phenotypes of Δ*tfoY* and Δ*lonA* deletions in WT, Δ2PDE, and Δ4DGC strains. (I) Overexpression of *tfoY* from the *Ptac* promoter in liquid culture and on plates (++) relative to uninduced (-). (J) Overexpression of *tfoY* from the *Ptac* promoter in liquid culture (+). Cells were then washed to remove the inducer and spotted onto plates lacking IPTG. T6SS-dependent killing was determined by enumerating the survival of *E*. *coli* strain MC4100, which is susceptible to T6SS attack. Statistical analysis was performed using an unpaired Student’s t-test. Statistical values indicated are (*p<0.05, **p<0.01, ***p < .001, and ****p < .0001).

Because c-di-GMP is known to regulate TfoY expression, we next explored whether TfoY abundance and stability is influenced by c-di-GMP levels *in vivo* [24,26]. We reasoned that DGCs and PDEs that impact *V*. *cholerae* motility could participate in the LonA-TfoY-c-di-GMP regulatory module and used strains lacking four diguanylate cyclases (Δ4DGC; Δ*cdgD* Δ*cdgH* Δ*cdgK* Δ*cdgL*) and two phosphodiesterases (Δ2PDE; Δ*rocS* Δ*cdgJ*) that impact *V*. *cholerae* motility to evaluate TfoY stability. We first assessed if natively produced TfoY would be detectable in WT, Δ*lonA*, Δ2PDE, and Δ4DGC strains using a Δ*lonA*Δ*tfoY* mutant as a negative control for TfoY production (Fig 5A). We were able to detect significant accumulation of TfoY in Δ*lonA*. In addition, we also observed the presence of TfoY in the Δ2PDE strain but not in WT, Δ4DGC, or Δ*lonA*Δ*tfoY* strains, suggesting that high levels of c-di-GMP in the Δ2PDE background stabilized TfoY, possibly by inhibiting LonA. Given that c-di-GMP can regulate the production of TfoY at both transcriptional and post-transcriptional levels, we assessed TfoY abundance in WT, Δ*lonA*, Δ*lonA*Δ2PDE, and Δ*lonA*Δ4DGC mutants to better understand how the LonA regulatory mechanism functions to control cellular levels of TfoY (Fig 5B) [24,26]. In the absence of *lonA*, TfoY levels were increased in all backgrounds relative to WT. Notably, levels of TfoY were significantly enriched in the low c-di-GMP strain in the absence of *lonA*. It was previously shown that c-di-GMP limits production of TfoY protein by binding to a riboswitch (Vc2) located in the 5’UTR of *tfoY* mRNA [24,27,28]. Thus, this result is consistent with prior analyses, which have shown that decreasing levels of c-di-GMP results in enhanced TfoY translation, and suggests that LonA plays a more significant role in regulating levels of TfoY when c-di-GMP levels are low [24,26]. In addition, we also observed a small but consistent increase in TfoY in the Δ*lonA*Δ2PDE relative to the Δ*lonA* strain (Fig 5B, S1 Fig). Notably, levels of LonA did not significantly differ in WT, Δ2PDE, and Δ4DGC strains (Fig 5C), suggesting that LonA quantity is not regulated by c-di-GMP. To determine if LonA turnover of TfoY might be influenced by c-di-GMP, we introduced the *Tn7*::*Ptac-tfoY* construct into the Δ4DGC and Δ2PDE strains and assessed TfoY stability relative to WT (Fig 5D). In addition, we simultaneously assessed global levels of c-di-GMP from these strains when *tfoY* was overproduced. We determined that cellular c-di-GMP levels are decreased 4.4-fold in the Δ4DGC and increased 2.2-fold in the Δ2PDE strains (Fig 5E). Interestingly, we found that TfoY stability is enhanced and decreased in Δ2PDE and in Δ4DGC strains, respectively suggesting that TfoY stability is positively correlated with cellular c-di-GMP. Taken together, our data demonstrates that c-di-GMP mediated inhibition of LonA proteolysis is a central factor governing levels of TfoY.

### C-di-GMP levels govern LonA and TfoY-dependent regulation of motility and the T6SS

Given that LonA and c-di-GMP coordinate TfoY stability, we wondered how these two factors might function to influence TfoY-mediated phenotypes. We first assessed how the deletion of *tfoY* and of *lonA* in WT, Δ2PDE, and Δ4DGC strains would alter motility and T6SS-dependent killing (Fig 5F and 5H). We did not observe any changes in motility or T6SS-dependent killing in Δ*tfoY*, Δ*tfoY* Δ2PDE, and Δ*tfoY* Δ4DGC strains relative to WT, Δ2PDE and Δ4DGC strains (Fig 5F and 5H). We also did not observe altered motility in Δ*lonA*Δ2PDE relative to the Δ2PDE strain or Δ*lonA*Δ4DGC relative to the Δ4DGC strain (Fig 5F). Both Δ*lonA* and Δ*lonA*Δ2PDE exhibited increased T6SS-dependent killing relative to WT; however, T6SS-dependent killing was not different between these strains (Fig 5F). In contrast, the Δ*lonA*Δ4DGC strain exhibited increased T6SS-dependent killing relative to Δ*lonA* and Δ*lonA*Δ2PDE strains (Fig 5F). Collectively, these results show that LonA regulates T6SS-dependent killing in WT, Δ2PDE, and Δ4DGC strains and that LonA-dependent regulation is most prominent when cellular c-di-GMP levels are low. These T6SS-dependent killing phenotypes correlate with the levels of TfoY observed in these strains (Fig 5E).

We next explored how overproduction of TfoY would impact motility and T6SS-dependent killing (Fig 5G and 5I). We analyzed these phenotypes in Δ*tfoY*, Δ*tfoY*Δ2PDE, and Δ*tfoY*Δ4DGC strains containing the *Tn7*::*Ptac-tfoY* construct. As expected, overexpression of *tfoY* led to increased motility in the Δ*tfoY* background (Fig 5G) [24]. Overproduction of TfoY also enhanced motility in the Δ*tfoY*Δ2PDE strain but not in the Δ*tfoY*Δ4DGC strain. It is noteworthy that the increase in TfoY-induced motility was 1.7-fold in Δ*tfoY*, 3.3-fold in Δ*tfoY*Δ2PDE, and 1.1-fold in Δ*tfoY*Δ4DGC strains. Together, these data suggest that as cellular levels of c-di-GMP increase, TfoY is able to play a more pronounced role in regulating motility. To analyze the impact of TfoY overproduction on T6SS-dependent killing, we induced TfoY production in cells grown in liquid cultures and on agar plates (Fig 5I). Overproduction of TfoY led to increased killing in all strains tested (Fig 5I). We observed greater T6SS-dependent killing in Δ*tfoY*Δ2PDE and Δ*tfoY*Δ*lonA* strains, conditions where TfoY is more stable and accumulates to higher levels (Fig 5A, 5B and 5D and S2A and S2B Fig). However, we also observed a significant increase in T6SS-dependent killing when TfoY was overproduced in the Δ*tfoY*Δ4DGC strain. The increase in T6SS-dependent killing was 26-fold in Δ*tfoY*, a 1,575-fold in Δ*tfoY*Δ2PDE, a 13,336-fold Δ*tfoY*Δ4DGC, and 1,944-fold Δ*tfoY*Δ*lonA* strain. The large increase in TfoY-dependent killing of *E*. *coli* in the Δ*tfoY*Δ4DGC strain was unexpected given that TfoY accumulates to substantially lower levels in this strain relative to the others tested (S2A and S2B Fig).

To determine if the increase in T6SS-dependent killing could be mediated by increased transcription of T6SS gene clusters, we assessed TfoY-mediated transcriptional activation of the T6SS by introducing transcriptional reporters of the large T6SS operon (*vipA-lux)* and of auxiliary cluster 2 (*hcp2-lux*) into the TfoY overproduction strains. TfoY overproduction led to increased T6SS gene expression in each of these strains (S3A and S3B Fig). However, we only observed very small increases in T6SS promoter activity between Δ*tfoY*Δ2PDE and Δ*tfoY*Δ4DGC strains relative to the Δ*tfoY* strain (S3A and S3B Fig). Such modest increases in transcription from these promoters are unlikely to account for the substantial increases in T6SS-dependent killing observed.

To further evaluate the impact of LonA and c-di-GMP regulation of TfoY stability on T6SS-dependent killing, we modified our experimental conditions in such a way that pools of TfoY were controlled before the onset of the T6SS-dependent killing assay. To do this, we first overproduced TfoY in Δ*tfoY*, Δ*tfoY*Δ*lonA*, Δ*tfoY*Δ2PDE, and Δ*tfoY*Δ4DGC strains in liquid-grown culture. We then collected cells and resuspend them in a buffer without inducer; the T6SS-dependent killing experiment were then performed on agar plates without inducer (Fig 5J). Overproduction of TfoY in the Δ*tfoY*Δ2PDE strain resulted in increased T6SS-dependent killing compared to Δ*tfoY* or Δ*tfoY*Δ4DGC strains (Fig 5J). However, in the Δ*lonA*Δ*tfoY* strain killing was restored to levels comparable to the Δ*tfoY*Δ2PDE strain (Fig 5J), indicating that LonA is the governing factor regulating TfoY-induced T6SS-dependent killing under the conditions tested.

### LonA activity can be directly regulated by c-di-GMP

**Fig 6:**
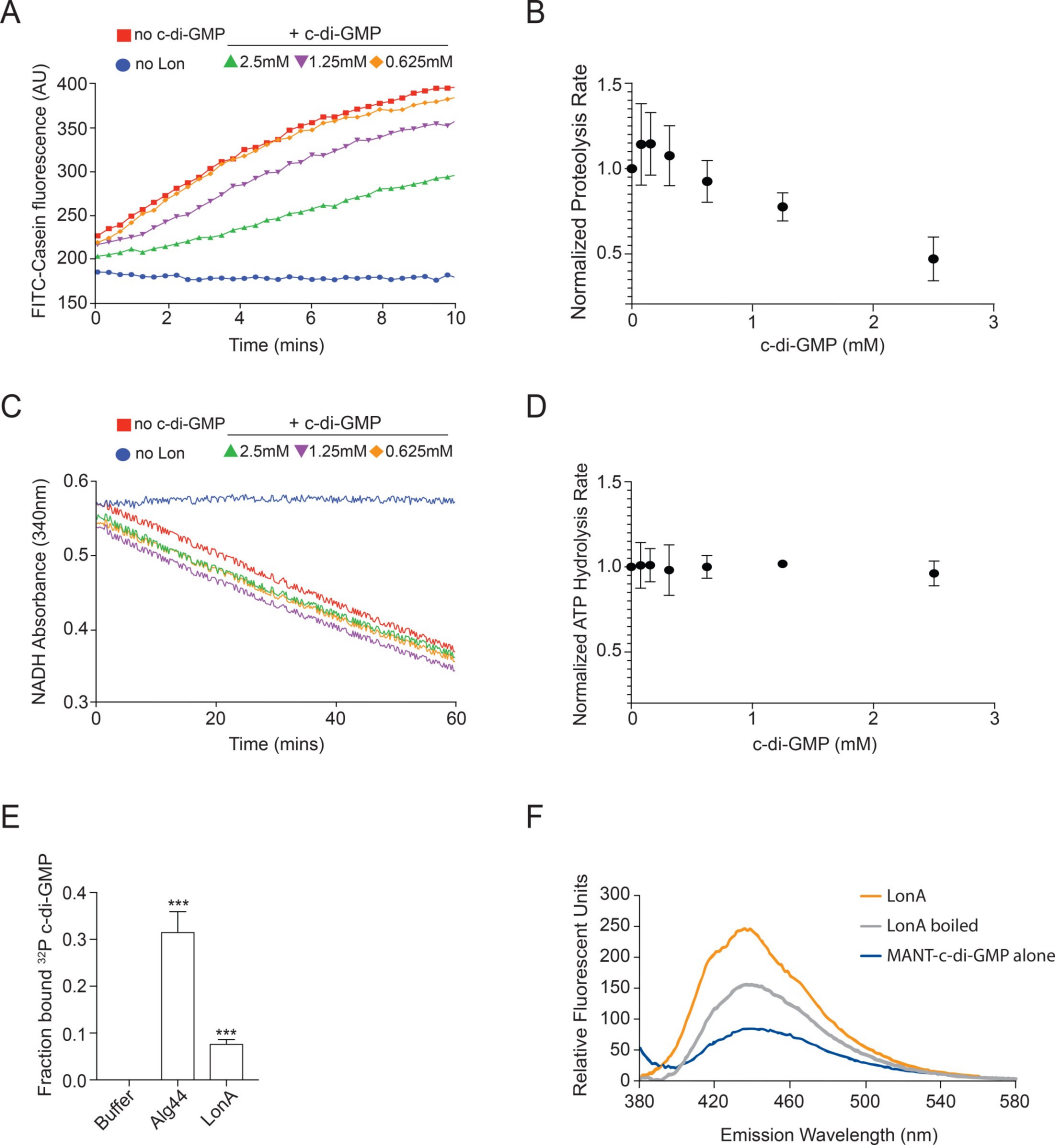
LonA proteolysis is directly regulated by c-di-GMP. *In vitro* proteolysis and protein ligand binding assays are shown. (A) Proteolysis of FITC-casein by purified LonA results in increased fluorescent signal. (B) Initial rate of substrate degradation as a function of c-di-GMP. (C) ATP hydrolysis for LonA alone and in the presence of c-di-GMP was monitored by loss of NADH, which is consumed stoichiometrically with ATP. (D) Rate of ATP hydrolysis as a function of c-di-GMP. Representative curves for (A) and (C) are shown with different concentrations of c-di-GMP. (E) Quantification of fraction bound of ^32^P-c-di-GMP to LonA by DRaCALA. Binding data shown represent the average and SD of triplicate independent experiments. Fraction bound by LonA and Alg44 were compared to buffer control by Student’s t-test. *** indicate p value of 0.0005, respectively. (F) Purified LonA or heat denatured Lon (boiled) were incubated with MANT-c-di-GMP. Representative emission fluorescence spectrum (excitation at 355 nm) is shown. Emission spectrum of MANT-c-di-GMP alone is also shown as a control.

Prior work has shown that c-di-GMP can inhibit Lon protease activity *in vitro*; however, this work was restricted to *E*. *coli* Lon [19]. Based on our *in vivo* work, we hypothesized that c-di-GMP may also function to reduce LonA activity in *V*. *cholerae*. In order to test this, we purified LonA and assessed how c-di-GMP influences LonA proteolysis of the model substrate casein. We found that addition of c-di-GMP substantially reduced the ability of LonA to degrade casein with approximately 60% reduction in activity at the highest c-di-GMP concentration tested (Fig 6A and 6B) with no effects on ATP hydrolysis in the conditions used (Fig 6C and 6D). We next tested direct binding of c-di-GMP, using a radiolabeled nucleotide DRaCALA assay and found that purified LonA bound c-di-GMP (Fig 6E), albeit with weak affinity [29]. We confirmed this result by using a c-di-GMP derivative (MANT-c-di-GMP) that increases fluorescence when bound [30]. Fluorescence of MANT-c-di-GMP increased when incubated with LonA and less so with heat denatured LonA, suggesting that a structured motif of LonA is likely important for c-di-GMP binding (Fig 6F). Taken together, these data show that LonA can weakly bind c-di-GMP directly and, most importantly for our model of TfoY regulation, that the *V*. *cholera* LonA protease activity can be inhibited by c-di-GMP.

## Discussion

LonA is a pleiotropic regulator in *V*. *cholerae* governing diverse behaviors such as cell morphology, biofilm formation, motility, c-di-GMP pools, the T6SS, virulence gene expression, and intestinal colonization [4]. In the current study, we fill significant gaps regarding the mechanism by which LonA controls these processes. We identified the T6SS and motility regulator TfoY as a LonA substrate and demonstrated that LonA-mediated proteolysis of TfoY functions to temper *V*. *cholerae’s* activation of flagellar mediated motility and T6SS-dependent killing. We determined that c-di-GMP reduces LonA-dependent proteolysis of a model substrate *in vitro*. In addition, we observed that cellular levels of TfoY are enhanced and stable when cellular c-di-GMP levels are high, providing evidence that c-di-GMP inhibits LonA proteolysis *in vivo*. Furthermore, we show how LonA and TfoY influence motility and T6SS-dependent killing phenotypes differently in strains with high and low levels of c-d-GMP relative to WT.

Prior to this study only two LonA substrates had been identified in *V*. *cholerae* [8,23]. The first is the alternative sigma factor FliA, which helps coordinate the activation of late stage flagellar genes and also acts to repress virulence factor production [8,31]. Upon assembly of the hook, or shearing of the flagellar filament, the anti-sigma factor FlgM is secreted through the flagella, permitting FliA to activate the genes necessary for flagellar function and the repression of virulence gene expression [8,31,32]. The release of FliA by FlgM, however, also causes FliA to become highly unstable due to proteolysis by LonA [8]. Thus, LonA’s control of FliA in the host may be important for derepressing virulence factor production. The second substrate is the quorum sensing master regulator HapR, which is proteolyzed by LonA during heat shock in order to activate biofilm formation [23]. We note that neither FliA nor HapR were identified in our proteome analysis, however, this is likely due to the physiological conditions required for LonA proteolysis of these regulators.

TfoY belongs to a class of proteins that contain TfoX-like N- and C-terminal domains. Proteins with TfoX-like domains are transcription factors that are frequently found in gamma-proteobacteria and are involved in the regulation of natural competency, the T6SS, and motility. Homologues of TfoY, such as TfoX of *V*. *cholerae*, Sxy-1 of *Haemophilus influenzae*, and Sxy of *E*. *coli* are regulators of DNA uptake and natural transformation in their respective organisms [24,33–36]. Prior studies have shown that TfoY does not control competency; TfoY is, however, an activator of motility in diverse Vibrio species and frequently controls T6SS-dependent killing as well [24,37]. It is important to note that the stability of Sxy of *E*. *coli* is also dependent upon LonA, which suggests that LonA proteolysis of TfoX-like domain containing proteins may be a common regulatory mechanism in diverse bacterial species [33].

While we did not observe TfoY to play a role in modulating an increase or decrease in c-di-GMP levels in the Δ*lonA* strains, transcriptomic analysis revealed that TfoY overproduction leads to significant transcriptional changes in a wide array of DGCs and PDEs [24]. Thus, it is possible that under certain conditions TfoY functions to alter levels of c-di-GMP within the cell by modulating the production of specific DGCs and PDEs. Of the c-di-GMP enzymes regulated by TfoY, the motility repressor CdgD appears to be the most significantly impacted [24,38–40]. Specifically, overproduction of TfoY leads to significant repression of *cdgD* [24]. Notably, we found that CdgD levels were elevated in WT relative to the *lonA* mutant. Thus, elevated TfoY in the *lonA* mutant may function to lower levels of CdgD in the cell. It is therefore possible that the enhanced motility observed in the *lonA* mutant and in *tfoY* overexpressing strains is at least in part due to repression of *cdgD*.

**Fig 7:**
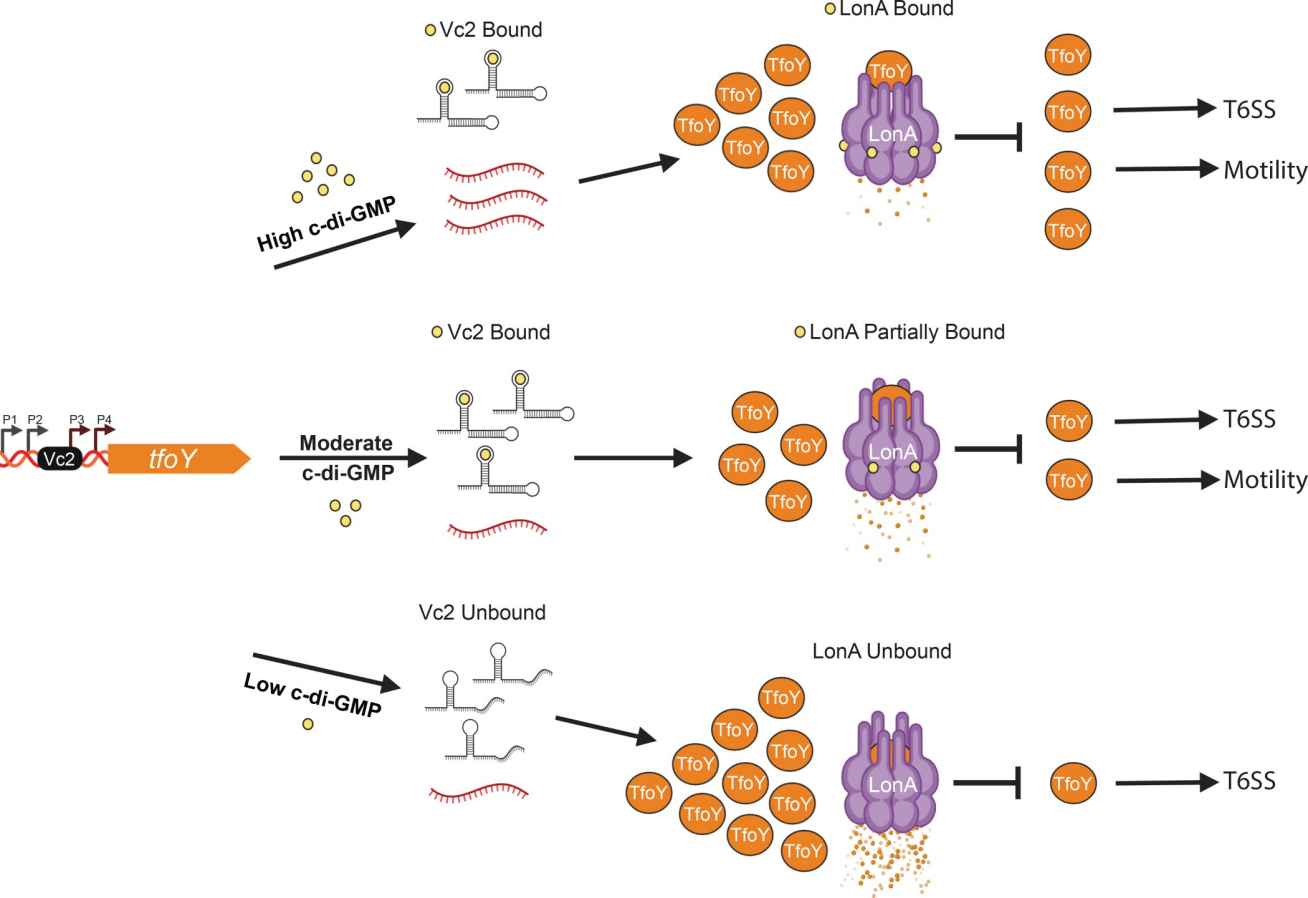
Model of LonA and c-di-GMP regulation of TfoY. Regulation of *tfoY* by c-di-GMP takes place at multiple regulatory points. TfoY production is regulated at the transcriptional level via a c-di-GMP binding transcriptional factor; at the post-transcriptional level via a c-di-GMP dependent riboswitch; and the post-translational level via c-di-GMP modulated proteolysis. The upstream regulatory region of *tfoY* contains 4 promoters. Promoters P1 and P2 (black bent arrows) produce transcript that contains the Vc2 riboswitch (close stem loop), which functions as an off switch when bound by c-di-GMP (yellow circle) and prevents translation [24,26,28]. In contrast, transcripts produced from promoters P3 and P4 (red bent arrows), driven by the c-di-GMP binding transcriptional activator VpsR, do not contain the Vc2 riboswitch [26]. In this model, we present our current understanding of post-transcriptional and post-translational regulation of TfoY (orange circles) by LonA (purple barrel) and c-di-GMP. At high c-di-GMP levels, transcripts without the Vc2 riboswitch accumulate at high levels. Transcript containing the Vc2 riboswitch are not translated. This results in moderate levels of TfoY. At the same time, high levels of c-di-GMP greatly reduce LonA-dependent proteolysis of TfoY, which results in TfoY activation of motility and T6SS-dependent killing. At intermediate c-di-GMP concentrations, transcripts with and without the Vc2 aptamer are present and LonA activity is reduced. This leads to low levels of TfoY production and the activation of motility and T6SS-dependent killing. At low c-di-GMP conditions, transcripts with the Vc2 aptamer are not inhibited by c-di-GMP. In addition, LonA activity is also not repressed by c-di-GMP, thereby leading to increased TfoY degradation. Under these conditions, TfoY activates T6SS-dependent killing but not motility.

While the environmental signals that govern the production of TfoY remain unclear, it has become increasingly apparent that sustained sensing of c-di-GMP by multiple c-di-GMP receptors is critical in controlling TfoY synthesis and turnover (Fig 7) [24,26,27,37,41–43]. For example, TfoY is regulated at the transcriptional level via a c-di-GMP binding transcriptional factor; at the post-transcriptional level via a c-di-GMP dependent riboswitch; and at the post-translational level via c-di-GMP modulated proteolysis. The upstream regulatory region of *tfoY* contains 4 promoters. Promoters P1 and P2 produce transcript that contains the Vc2 riboswitch, which restricts translation when bound by c-di-GMP [24,27,28]. Decreasing levels of c-di-GMP derepresses the Vc2 inhibitory mechanism and permits translation of *tfoY* mRNA into TfoY protein [24,26–28]. However, elevated levels of c-di-GMP also permit the production of TfoY [26]. This occurs through the master regulator of biofilm formation, VpsR, which binds to *tfoY* promoter elements P3 and P4 within and downstream of the Vc2 riboswitch, respectively, resulting in transcript lacking the Vc2 inhibitory mechanism [26]. It is important to note that decreasing levels of c-di-GMP leads to greater production of TfoY than elevating c-di-GMP does [24,26,37]. We note that these studies utilized TfoY fluorescent fusions to evaluate TfoY production. It has been previously shown that fluorescent fusions with sfGFP can severely limit LonA proteolysis unless circular permuted variants of sfGFP are used [44]. Thus, the translational reporters utilized in prior studies may have prevented LonA-mediated proteolysis of TfoY, and would therefore reflect TfoY protein production but not TfoY turnover [24,26,37]. Consistent with this hypothesis, we observe that a greater amount of TfoY accumulates in the Δ4DGC strain relative to the Δ2PDE strain in mutants where *lonA* has been deleted.

Our findings suggest the presence of an additional regulatory module governing TfoY stability in a c-di-GMP dependent manner. Specifically, when c-di-GMP levels are decreased, the Vc2 regulatory mechanism is bypassed and TfoY protein is produced efficiently. However, because LonA is unimpeded by c-di-GMP, it proteolyzes TfoY efficiently as well. On the other hand, when c-di-GMP levels are high, TfoY production occurs less efficiently and LonA proteolysis of TfoY is similarly scaled back by c-di-GMP. Therefore, the same ligand that drives large changes in transcriptional and translational control of TfoY expression also buffers steady state levels of TfoY through proteolysis.

Our genetic and phenotypic analyses also suggest that TfoY may play different roles under high and low c-di-GMP conditions. For example, we observed that TfoY is increasingly important in driving motility when c-di-GMP levels are increased. Levels of c-di-GMP are elevated in biofilms, thus it is possible that TfoY may aid in dispersal from biofilms by enhancing motility. Conversely, we did not observe TfoY to significantly influence motility when c-di-GMP levels were low, which is consistent with a prior analysis that analyzed TfoY-dependent motility when a phosphodiesterase was overproduced [24]. TfoY also enhances T6SS-dependent killing when c-di-GMP levels are increased, indicating that TfoY may have a role in mediating T6SS-dependent competition in biofilms. Similarly, overproduction of TfoY resulted in robust T6SS-dependent killing in strains with low cellular levels of c-di-GMP. We found that the increases in T6SS-dependent killing in strains with high and low c-di-GMP levels are likely independent of TfoY’s ability to activate transcription from the two T6SS operons tested. Thus, it is possible that TfoY functions synergistically with additional c-di-GMP dependent regulatory factors to enhance *V*. *cholerae* fitness against its competitors. An intriguing observation from this analysis is that overproduction of TfoY led to the most significant T6SS-dependent killing phenotype in a strain with low cellular levels of c-di-GMP, despite levels of TfoY also being lowest under in this background. While the physiological significance of this regulation remains to be fully elucidated, these findings suggests that TfoY is capable of mounting a particularly powerful T6SS-dependent assault when cellular c-di-GMP levels are reduced. It was previously hypothesized that activation of TfoY could be part of a danger sensing system utilized in a defensive escape response [24]. The results of our genetic and phenotypic analysis suggest that the production of TfoY could be important for launching a T6SS-mediated counter-attack in the presence of a c-di-GMP reducing “danger signal,” and that LonA and c-di-GMP would be central regulators controlling the magnitude and longevity of this output.

Our finding that c-di-GMP limits LonA proteolysis in *V*. *cholerae* is consistent with a previous report that found addition of c-di-GMP inhibits degradation of α-casein by *E*. *coli* Lon in an endpoint assay [19]. The same study also found that binding of c-di-GMP to Lon could be indirectly monitored by loss of HPLC signal [19]. In our current work, we extend upon those findings by using kinetic assays to monitor degradation in real-time. We also demonstrate c-di-GMP weakly binds to LonA using a radiolabeled assay and a fluorescent assay. It remains to be determined how LonA binds c-di-GMP, how selective this binding is, and how binding of this ligand reduces LonA activity. It will be important to assess how c-di-GMP modulates LonA proteolysis against a wide range of substrates in order to determine if c-di-GMP functions solely as a repressor or if it can also function to enhance proteolysis by altering LonA target specificity.

## Methods

### Ethics statement

All animal procedures used were in strict accordance with the *Guide for the Care and Use of Laboratory Animals* [45] and were approved by the University of California (UC), Santa Cruz, Institutional Animal Care and Use Committee, Santa Cruz, CA (approval number Yildf1206).

### Bacterial strains, growth conditions and antibody generation

The bacterial strains used in this study can be found in S4 Table. *V*. *cholerae* and *E*. *coli* strains were grown aerobically in Lysogeny Broth (LB) broth (1% tryptone, 0.5% yeast extract, 1% NaCl, pH 7.5) at 30°C and 37°C, respectively. LB agar contained granulated agar (Difco) at 1.5% (wt/vol). Antibiotics were used when necessary at the following concentrations: rifampin, 100 μg/ml; ampicillin, 100 μg/ml; gentamycin, 15 μg/ml; and chloramphenicol, 2.5ug/ml, 5ug/ml, or 100 μg/ml as indicated in the text.

### Strain and plasmid generation

Plasmids were constructed using standard cloning methods or the Gibson Assembly recombinant DNA technique (New England BioLabs, Ipswich, MA). Gene deletions were carried out using allelic exchange of the native open reading frame (ORF) with a truncated ORF, as previously described [39]. The generation of complementation and overexpressing mutants was carried out using a Tn7-based system, as previously described [4,25]. For the *tfoY* complementation construct, the open reading frame of *tfoY*, as well as 500bp upstream, were cloned into pGP704-Tn7 plasmid. For the *tfoY* overexpression construct, the open reading frame of *tfoY* was cloned into pMMB67EH, which contains the IPTG inducible *Ptac* system. The *Ptac-tfoY* fusion was then cloned into the pGP704-Tn7 plasmid. Triparental matings with donor *E*. *coli* S17λpir carrying the pGP704-Tn7 plasmid with the gene of interest, helper *E*. *coli* S17λpir harboring pUX-BF13, and *V*. *cholerae* deletion strains were carried out by mixing all three strains and incubating mating mixtures on LB agar plates for 18 h at 30°C. Transconjugants were selected on LB media containing rifampicin and gentamycin at 30°C. Insertion of the complementation construct to the Tn7 site was verified by PCR. *V*. *cholerae* WT and mutant strains were tagged with green fluorescent protein (GFP) according to a previously described procedure [46]. The GFP-tagged *V*. *cholerae* strains were verified by PCR and used in biofilm analyses. Transcriptional reporters were generated by cloning the upstream of the regulatory region as well as a portion of the open reading frame of VCA0107 (*vipA*) or VCA0017 (*hcp2*) into the pBBR*lux* plasmid using established methodologies [47]. The exact lengths of the regulatory region used can be found in the supplementary material (S4 Table).

### Whole proteome analysis

*V*. *cholerae* WT and Δ*lonA* strains that lacked the genes for cholera toxin (*ctxAB*) were grown aerobically at 30°C overnight. The cultures were diluted 1:500 and grown to OD_600_ = 1.0, at which point 100μg/mL of chloramphenicol was added to stop translation. After 1-hour of chloramphenicol treatment, cells were collected by centrifugation and flash frozen in liquid nitrogen, and then stored in -80°C. Five biological replicates for WT and Δ*lonA* were collected. These samples were processed at NYU Proteomics Facility for analysis by liquid chromatography coupled with mass spectrometry.

### Proteome extraction and sample preparation for mass spectrometry analysis

Cell pellets were resuspended in 8M urea in 100 mM HEPES buffer (pH 8.0) and 250μg of each protein lysates were reduced using dithiothreitol (5μl of 0.2 M) for 1 h at 55°C. The reduced cysteines were subsequently alkylated with iodoacetamide (5μl of 0.5 M) for 45 min in the dark at room temperature. Next, 20 mM HEPES (pH 8.0) were added to dilute the urea concentration to 2 M and the protein lysates were digested with Trypsin (Promega) at a 100:1 (protein:enzyme) ratio overnight at room temperature. The pH of the digested protein lysates was lowered to pH < 3 using trifluoroacetic acid (TFA). The digested lysates were desalted using C18 solid-phase extraction (Sep-Pak, Waters). 40% acetonitrile (ACN) in 0.5% acetic acid followed by 80% acetonitrile (ACN) in 0.5% acetic acid was used to elute the desalted peptides. The peptide eluate was concentrated in the SpeedVac and stored at -80°C.

### Tandem Mass Tag (TMT) labeling

The dried peptide mixture was re-suspended in 100 mM TEAB (pH 8.5) using a volume of 100 μl. Isobaric mass tag labeling was performed used the TMT 10plex reagent set from ThermoFisher. Each sample was labeled with TMT reagent according to the manufacturer’s protocol. In brief, each TMT reagent vial (0.8 mg) was dissolved in 41 μL of anhydrous ethanol and was added to each sample. The reaction was allowed to proceed for 60 min at room temperature and then quenched using 8 μL of 5% w/v hydroxylamine. The samples were combined at a 1:1 ratio and the pooled sample was subsequently desalted using SCX and SAX solid-phase extraction columns (Strata, Phenomenex) as described [48].

### Global proteome analysis

A 500 μg aliquot of pooled sample was fractionated using basic pH reverse-phase HPLC using previously established procedures [49]. Briefly, the sample was loaded onto a 4.6 mm × 250 mm Xbridge C18 column (Waters, 3.5 μm bead size) using an Agilent 1260 Infinity Bio-inert HPLC and separated over a 70 min linear gradient from 10 to 50% solvent B at a flow rate of 0.5 ml/min (Buffer A  =  10 mM ammonium formate, pH 10.0; Buffer B  =  90% ACN, 10 mM ammonium formate, pH 10.0). A total of 40 fractions were collected throughout the gradient. The early, middle and late eluting fractions were concatenated and combined into 10 final fractions. The combined fractions were concentrated in the SpeedVac and stored at -80°C until further analysis.

### LC-MS/MS analysis

An aliquot of each sample was loaded onto a trap column (Acclaim PepMap 100 pre-column, 75 μm × 2 cm, C18, 3 μm, 100 Å, Thermo Scientific) connected to an analytical column (EASY-Spray column, 50 m × 75 μm ID, PepMap RSLC C18, 2 μm, 100 Å, Thermo Scientific) using the autosampler of an Easy nLC 1000 (Thermo Scientific) with solvent A consisting of 2% acetonitrile in 0.5% acetic acid and solvent B consisting of 80% acetonitrile in 0.5% acetic acid. The peptide mixture was gradient eluted into the QExactive mass spectrometer (Thermo Scientific) using the following gradient: a 5%-23% solvent B in 100 min, 23% -34% solvent B in 20 min, 34% -56% solvent B in 10 min, followed by 56%- 100% solvent B in 20 min. The full scan was acquired with a resolution of 70,000 (@ *m*/*z* 200), a target value of 1e6 and a maximum ion time of 120 ms. After each full scan 10 HCD MS/MS scans were acquired using the following parameters: resolution 35,000 (@*m*/*z* 200), isolation window of 1.5 *m*/*z*, target value of 1e5, maximum ion time of 250 ms, normalized collision energy (NCE) of 30, and dynamic exclusion of 30 s.

### Data analysis

Raw mass spectrometry data were processed using Proteome Discoverer 2.1. Proteins and peptides were searched against the PATRIC *Vibrio cholerae* using the Byonic with a protein score cut-off of 300, using the following settings: oxidized methionine (M), and deamidation (NQ) were selected as variable modifications, and carbamidomethyl (C) as fixed modifications; precursor mass tolerance 10 ppm; fragment mass tolerance 0.02 Da. The following filters and criteria were used for quantification: Proteins identified with less than two unique peptides were excluded from analysis. Bioinformatics analysis was performed with Perseus, Microsoft Excel and R statistical computing software. Student’s t-test using Benjamini-Hochberg FDR cutoff of 5% was then used to identify proteins that were differentially enriched.

### *In vivo* proteolysis and protein abundance assays

Overnight cultures of *V*. *cholerae* were diluted 1:500 in 100mL of LB medium. Cells were grown until an OD_600_ = 0.1 was reached, 0.1mM IPTG was added to induce transcription of *tfoY* from the *Ptac* promoter. Induction proceeded for 2-hours, 2mL aliquots were taken immediately prior, and then at the time points indicated after the addition of 100x the minimum inhibitory concentration of chloramphenicol (100μg/mL). Protein abundance assays were performed similarly with minor modifications noted in the figure legends. BCA analysis was used to quantify total protein loaded. Relative levels of TfoY, LonA, and RNAP were assessed by western blot analysis. The TfoY antibody was used at 1μg/mL concentration, the LonA antibody at 0.5μg/mL, and the RNAP antibody was used at 0.625μg/mL. At least three independent biological replicates were performed for all *in vivo* protein abundance and stability assays. For densitometric analysis, software from Image Lab v6.01 (Bio Rad Laboratories) was used to quantify band intensity of TfoY from western blots. Adjusted total band intensity was calculated by subtracting the background intensity values from the total band intensity value.

### Swimming motility assays

Flagellar motility was determined by inoculating a single overnight colony into the center of LB soft agar plates (0.3% wt/vol). For overexpression experiments, the soft agar plates were supplemented with 0.1mM IPTG to induce expression from the *Ptac* promoter. The plates were moved to 30°C and the swimming diameter was recorded 18-hours post inoculation. The motility phenotype of each mutant was assessed using at least three independent biological replicates. Statistical analysis of motility was performed using an unpaired Student’s t-test.

### Interbacterial killing assays

*V*. *cholerae* WT and mutant strains and the *E*. *coli* strain MC4100 were grown overnight in LB medium at 30°C and 37°C respectively. Overnight grown cultures of *V*. *cholerae* and *E*. *coli* strains were diluted 1:200 in LB medium supplemented with 340mM NaCl. *V*. *cholerae* strains were grown to an OD_600_ = 0.8–1.0 while *E*. *coli* was grown to an OD_600_ = 0.4–0.8. Approximately 10^9^ *V*. *cholerae* and 10^8^ *E*. *coli* cells were mixed and 25μL of this mixture was spotted in technical triplicate onto nitrocellulose membrane that had been placed on LB agar supplemented with 340mM NaCl. For overexpression experiments, 0.1mM IPTG was included in the liquid media and/or plates as indicated in the text. In the interbacterial competition assay that addressed TfoY stability on T6SS-dependent killing (Fig 5J), 0.1mM IPTG was included in liquid media, the cells were then washed twice with 1x PBS, and then *V*. *cholerae* and *E*. *coli* were mixed and spotted onto 340mM NaCl agar plates lacking IPTG. Interbacterial competition was allowed to proceed for approximately 4-hours at 37°C, at which point the filter membranes were removed and resuspended in 1mL of 1x PBS. Cells were resuspended and serial dilutions were generated and spotted onto LB plates containing 100μg/mL of streptomycin, grown overnight, and the surviving *E*. *coli* was enumerated. Statistical analysis of T6SS killing was performed using an unpaired Student’s t-test.

### Biofilm assays

Flow cell chambers were inoculated with 200μL of overnight-grown cultures of *gfp*-tagged *V*. *cholerae* strains that had been diluted to an OD_600_ of 0.02. Once inoculated, the bacteria were allowed to adhere at room temperature for 1 hr without flow. Next, the flow of 2% (vol/vol) LB (0.2 g/liter tryptone, 0.1 g/liter yeast extract, 1% NaCl) was initiated at a rate of 7.5 ml/h and continued for 24 h. Confocal laser scanning microscopy (CLSM) images of the biofilms were captured with the Zeiss 880 microscope using an excitation wavelength of 488 nm and an emission wavelength of 543 nm. Three-dimensional images of the biofilms were reconstructed and analyzed using Imaris software (Bitplane). Biofilm analysis was performed using COMSTAT2. Statistical analysis of biofilms was performed with ANOVA, utilizing Tukey’s multiple comparison analysis.

### Quantification of Intracellular c-di-GMP

*V*. *cholerae* WT and mutant strains were grown to an OD_600_ = 0.4 in LB medium at 30°C, at which point 40mL of culture was spun down for quantification of c-di-GMP as previously described [39]. In addition, 4mL of culture was spun down for determination of protein concentration via BCA. Quantification of c-di-GMP in each sample was performed by comparing values to a standard curve generated with pure c-di-GMP that had been resuspended in 184mM NaCl.). Three independent biological replicates were analyzed. Statistical analysis of c-di-GMP pools was performed with ANOVA, utilizing Tukey’s multiple comparison analysis.

### Intestinal colonization assays

Colonization of the infant mouse was performed as previously described [4]. Briefly, a WT strain, a WT strain lacking *lacZ* (Δ*lacZ*), Δ*lonA*, Δ*tfoY*, and Δ*lonA* Δ*tfoY* mutants were grown overnight at 30°C in LB media. The mutant strains (*lacZ*^+^) strains were competed against otherwise WT Δ*lacZ* strain at a 1:1 ratio in 1xPBS containing Evan’s blue dye. The input inoculum was serial diluted and plated onto LB agar plates supplemented with X-gal such that *lacZ* containing strains could be visually differentiated from the Δ*lacZ* WT. Approximately 10^5^ CFU were intragastrically administered to groups of 5-day-old CD-1 mice (Charles River Laboratories, Hollister, CA). At 20hrs post infection, the small intestine was removed, weighed, and homogenized. The ratio of the Δ*lacZ* WT and the respective competing strains were determined by serial diluting and plating the homogenized intestine onto LB agar plates containing rifampicin and X-gal. Statistical analyses were performed using the Wilcoxon’s signed-ranked test.

### DRaCALA measurment of ligand binding

For binding assay, final concentrations of the following are used: 2 μM Vc Lon was mixed with in 1x binding buffer (10 mM Tris, pH 8.0, 100 mM NaCl) and added to 3.3 nM ^32^P-c-di-GMP in a 20 μL reaction. The entire reaction was incubated for 1 min at room temperature and 2 μL of the reaction was applied to dry nitrocellulose paper to perform DRaCALA. Once dried and imaged, the fraction bound quantified using Fujifilm Multi Gauge software v3.0. The assays were performed in triplicate [29].

### Measurement of MANT-c-di-GMP binding

MANT-c-di-GMP (2'-O-(N'-Methylanthraniloyl)-cyclic diguanosine monophosphate; BioLog, Germany) was added at 2.5 μM to LonA at 0.7 μM hexamer either native or denatured by heating at 95°C for 20 minutes. Fluorescence was measured by excitation at 355 nm and scanning emission from 380–600 nm. Assays were performed in triplicate [30].

### Protein purification and *in vitro* proteolysis assays

Full length *lonA* from *V*. *cholerae* was cloned into a pBAD33 expression vector and transformed into BL21DE3. Overnight cultures grown at 37°C were back diluted into 6 L of LB + 100 μg/ml ampicillin, grown to mid-log phase, and induced by addition of 0.2% L-arabinose for 3 hours. Cells were pelleted by centrifugation then pellets were resuspended in lysis buffer (100 mM potassium phosphate at pH 6.5, 1 mM DTT, 1 mM EDTA, 10% glycerol). Following microfluidization disruption, the lysate was clarified by centrifugation (15,000 g / 30 minutes), and supernatant was applied to 10 ml of washed hydroxyapatite resin (Sigma). Following batch binding for 1 hour at 4°C, bound resin was washed twice (3 column volumes each) with buffer A (100 mM potassium phosphate at pH 6.5, 1 mM DTT, 1 mM EDTA, 10% glycerol), then twice (3 column volumes each) with buffer B (200 mM potassium phosphate at pH 6.5, 1 mM DTT, 1 mM EDTA, 10% glycerol), centrifuging at 2000 g for 10 minutes following each wash to separate the resin. Protein was eluted by 3 washes (1 column volume each) with elution buffer (400 mM potassium phosphate at pH 6.5, 1 mM DTT, 1 mM EDTA, 10% glycerol). Elutions were filtered using 0.22 um filters, then concentrated to 5 ml using centrifugal concentrators (10 kDa cutoff). Concentrate was loaded onto a Sephacryl S-200 (120 ml) equilibrated in 50 mM Tris (pH 8), 1 mM DTT, 1 mM EDTA, and 20% glycerol and eluted with the same buffer. Activity was monitored by testing fractions using FITC-casein (see below). Active fractions were pooled and loaded onto a 1ml Mono-Q equilibrated in Qbuffer A (25 mM Tris pH 8, 50 mM KCl, 10% glycerol, 1 mM DTT), washed extensively and eluted with a 30 CV (0–100%) gradient using Qbuffer B (25 mM Tris pH 8, 1M KCl, 20% glycerol, 1 mM DTT). Final active fractions were concentrated, snap frozen in liquid nitrogen, and stored at -80°C. Standard proteolysis reactions contain 100–200 nM LonA (hexamer concentration) in degradation buffer (20 mM Tris pH 8, 100 mM KCl, 10 mM MgCl2) with 4 mM ATP, 5 mM creatine phosphate, 7.5 ug/ml creatine kinase (for ATP regeneration) and 10 μg/ml FITC-Casein (Sigma). Increases in fluorescence (ex 460 nm / em 520 nm) due to degradation of the labeled substrate was monitored using a Spectramax M5 (Molecular Devices) in 384-well nonbinding surface plates (Corning) incubated at 30°C. ATP hydrolysis was monitored using a coupled NADH-based assay where loss of NADH corresponds 1:1 with hydrolysis of ATP [50].

### Analysis of promoter activity of T6SS operons

Luminescence assays were performed as previously described with minor alterations [4,38,51]. Briefly, overnight cultures of *V*. *cholerae* were grown in LB media containing 5μg/mL of chloramphenicol. Cells were diluted 1:500 in LB media containing 2.5μg/mL chloramphenicol and grown to late exponential phase (OD_600_ ~1.0). Cells were diluted 1:10 in LB and luminescence was measured in technical triplicate. Relative luminescence was quantified across at least 3 biological replicates. Statistical analysis was performed using an unpaired Student’s t-test.

## Supporting information

S1 Fig Levels of TfoY are elevated in the Δ*lonA*Δ2PDE and Δ*lonA*Δ4DGC strains relative to Δ*lonA*. Semiquantitative densitometric analysis from western blots shown in Fig 5B. Levels of TfoY from WT, Δ*lonA*, Δ*lonA*Δ2PDE, and Δ*lonA*Δ4DGC mutants were analyzed using Image Lab. Shown are the arbitrary intensity values from two independent biological replicates.
(TIF)

S2 Fig Levels of TfoY are elevated in the Δ2PDE and Δ*lonA* strains prior to the beginning of T6SS killing experiments. *In vivo* abundance of TfoY was analyzed in Δ*tfoY*, Δ*tfoY*Δ2PDE, and Δ*tfoY*Δ4DGC mutant strains before mixing *V*. *cholerae* with *E*. *coli* in the T6SS-dependent killing experiment described in Fig 5H. (A) Cells were either grown in the absence of IPTG (-) or (B) in the presence of IPTG (+) to overexpress *tfoY* from the *Ptac* promoter. Levels of TfoY were analyzed by western blot using the αTfoY antibody. RNAP was used as a control for sample loading in all western blots.
(TIF)

S3 Fig Overproduction of TfoY on T6SS gene expression in different strains with cellular c-di-GMP levels. The impact of TfoY on T6SS gene expression phenotypes was assessed in Δ*tfoY*, Δ*tfoY*Δ2PDE, and Δ*tfoY*Δ4DGC strains harboring T6SS gene transcriptional reporters for either the (A) regulatory region upstream of *vipA* or (B) the regulatory region upstream of *hcp2*. Cells were either grown in the absence of IPTG (-) or in the presence of IPTG (+) to overexpress *tfoY* from the *Ptac* promoter. Bioluminescence was assessed at late exponential phase. Statistical analysis was performed using an unpaired Student’s t-test. Statistical values indicated are (*p<0.05, **p<0.01, and ****p < .0001).
(TIF)

S1 Data Raw data used for all graphs included in this work. (XLSX)

S1 Table Proteins enriched in Δ*lonA* relative to WT. (PDF)

S2 Table Proteins enriched in WT relative to Δ*lonA*. (PDF)

S3 Table Analysis of WT, Δ*tfoY*, Δ*lonA*, and Δ*lonA*Δ*tfoY* biofilms. (PDF)

S4 Table Strains and plasmids used in this study. (PDF)
