# Supplementary figures and images for: c-di-GMP inhibits LonA-dependent proteolysis of TfoY in Vibrio cholerae
Source: PLoS Genet. 2020 Jun 26;16(6):e1008897. doi: 10.1371/journal.pgen.1008897 (PMC7371385; doi:10.1371/journal.pgen.1008897)

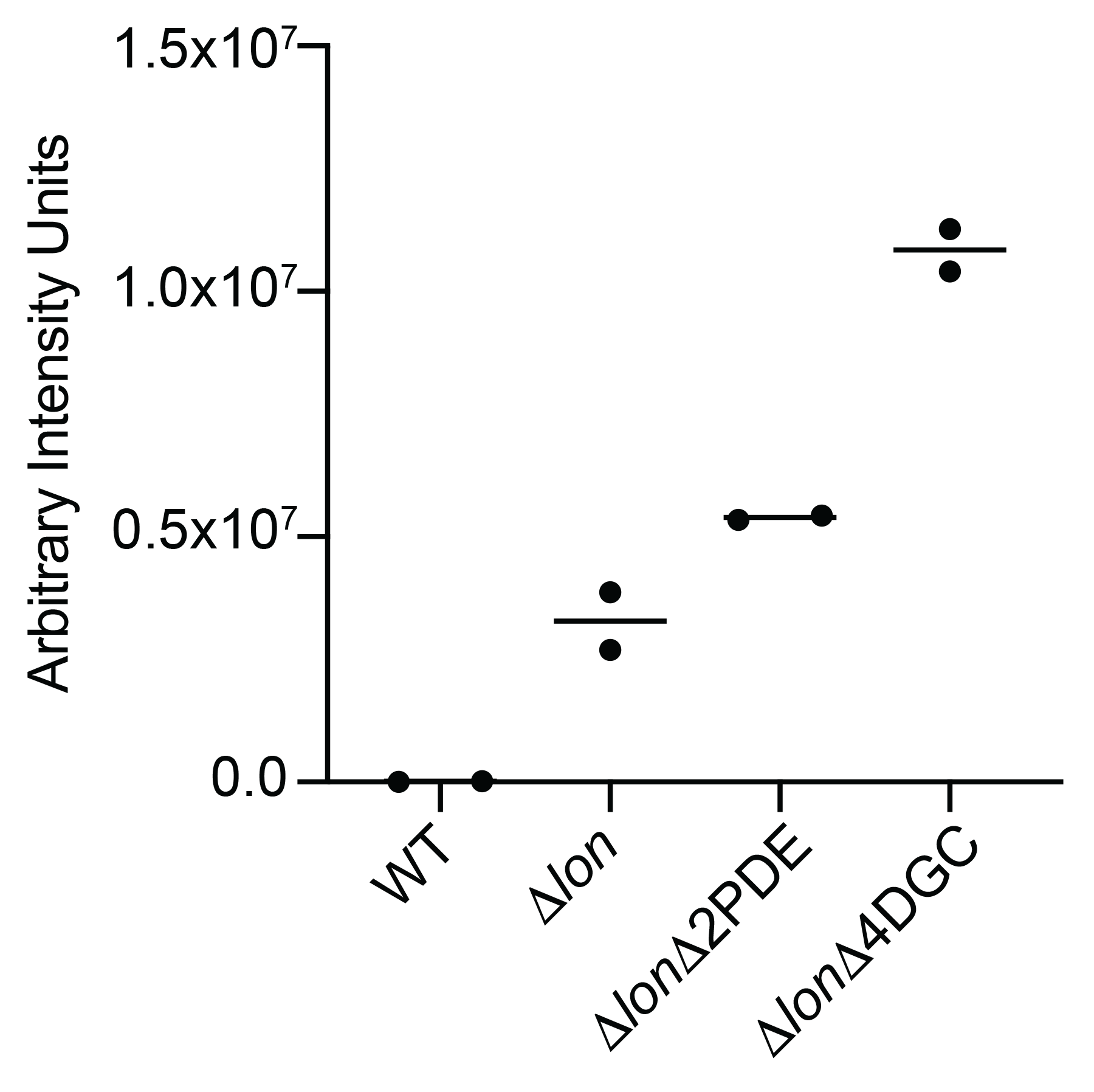

Supplement: S1 Fig — Semiquantitative densitometric analysis from western blots shown in Fig 5B. Levels of TfoY from WT, ΔlonA, ΔlonAΔ2PDE, and ΔlonAΔ4DGC mutants were analyzed using Image Lab. Shown are the arbitrary intensity values from two independent biological replicates. (TIF) [file pgen.1008897.s001.tif]

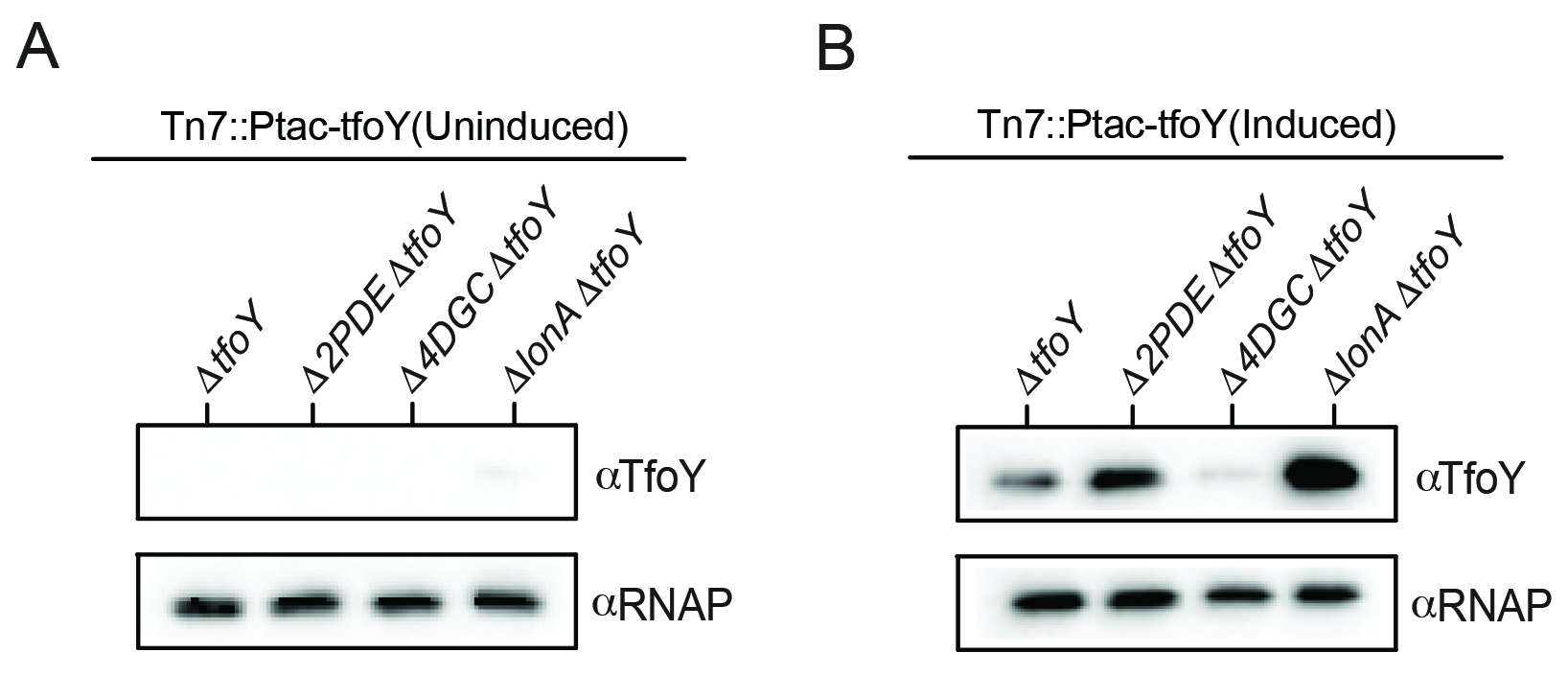

Supplement: S2 Fig — In vivo abundance of TfoY was analyzed in ΔtfoY, ΔtfoYΔ2PDE, and ΔtfoYΔ4DGC mutant strains before mixing V. cholerae with E. coli in the T6SS-dependent killing experiment described in Fig 5H. (A) Cells were either grown in the absence of IPTG (-) or (B) in the presence of IPTG (+) to overexpress tfoY from the Ptac promoter. Levels of TfoY were analyzed by western blot using the αTfoY antibody. RNAP was used as a control for sample loading in all western blots. (TIF) [file pgen.1008897.s002.tif]

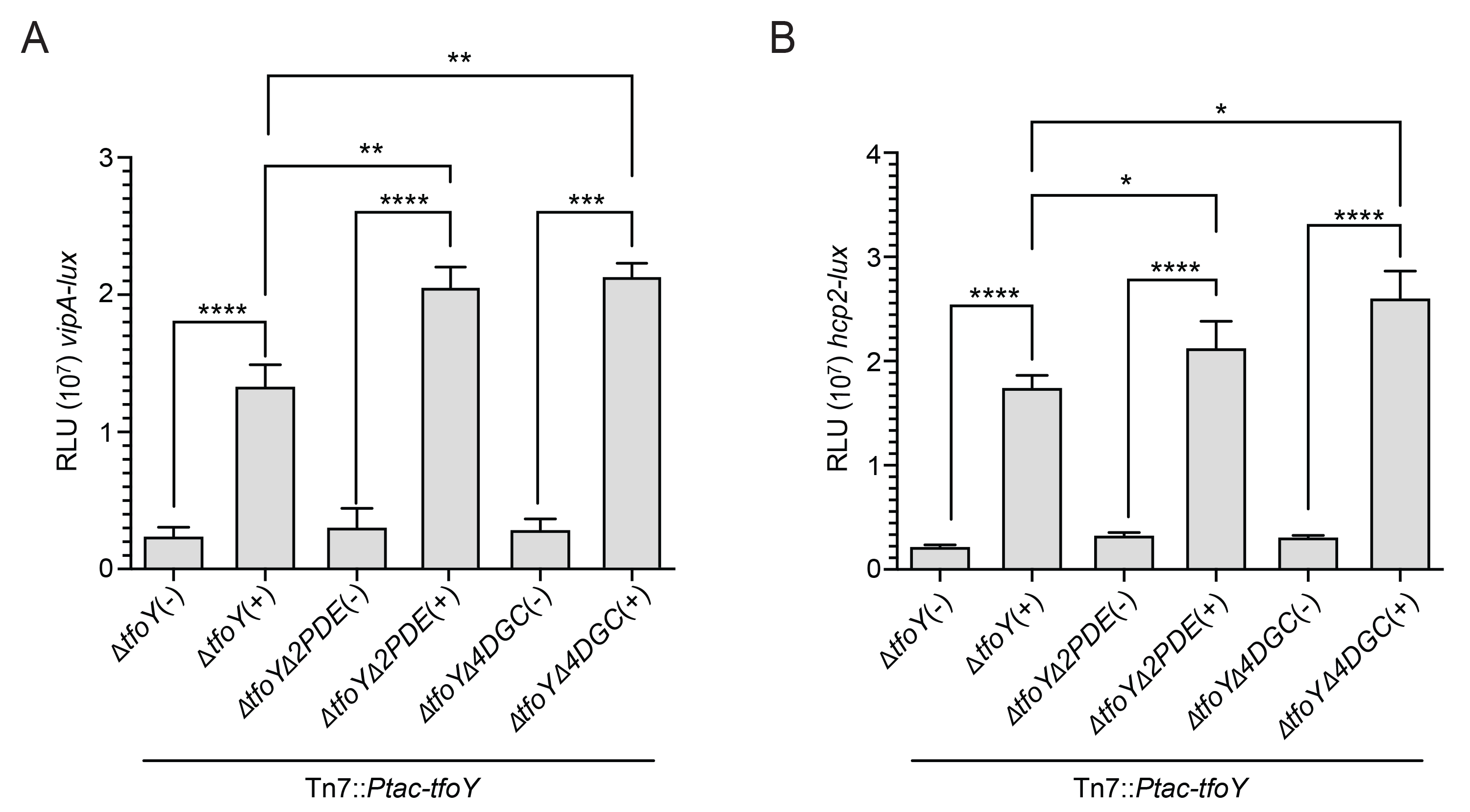

Supplement: S3 Fig — The impact of TfoY on T6SS gene expression phenotypes was assessed in ΔtfoY, ΔtfoYΔ2PDE, and ΔtfoYΔ4DGC strains harboring T6SS gene transcriptional reporters for either the (A) regulatory region upstream of vipA or (B) the regulatory region upstream of hcp2. Cells were either grown in the absence of IPTG (-) or in the presence of IPTG (+) to overexpress tfoY from the Ptac promoter. Bioluminescence was assessed at late exponential phase. Statistical analysis was performed using an unpaired Student’s t-test. Statistical values indicated are (*p<0.05, **p<0.01, and ****p < .0001). (TIF) [file pgen.1008897.s003.tif]
