# Supplementary material for: c-di-GMP inhibits LonA-dependent proteolysis of TfoY in Vibrio cholerae
Source: PLoS Genet. 2020 Jun 26;16(6):e1008897. doi: 10.1371/journal.pgen.1008897 (PMC7371385; doi:10.1371/journal.pgen.1008897)
Supplement: S1 Table — (PDF) [file pgen.1008897.s005.pdf]

Joshi et al.

S1 Table. Proteins enriched in Δ*lonA* relative to WT[a]

Gene	Description	Fold Change (log2)
VC1722	T6SS and motility regulator; TfoY	4.289727879
VC0049	hypothetical protein	1.289696121
VCA0422	hypothetical protein	0.922209424
VCA1069	methyl-accepting chemotaxis protein	0.783467412
VCA0540	formate transporter 1, putative	0.693574789
VCA0349;VCA0504	relB protein	0.688476652
VC0018	16 kDa heat shock protein A	0.66216644
VCA0107	T6SS outer tube; vipA	0.646651164
VC2240	decarboxylase	0.641421098
VCA0271	hypothetical protein	0.607169032
VCA0933	cold shock domain-contain protein; cspV	0.580625454
VC0715	nitroreductase A	0.570175745
VCA0281	integrase, putative	0.568173608
VCA0248	putative L-ascorbate 6-phosphate lactonase	0.544823691
VC2296	bolA protein	0.509163114
VC0092	LexA repressor	0.508418256
VC2735	hypothetical protein	0.500998747
VC2664	chaperonin GroEL	0.455176759
VC2675	ATP-dependent protease peptidase subunit; HslV	0.443346247
VC0945	hypothetical protein	0.437054759
VCA0752	thioredoxin 2	0.410689113
VCA0020	T6SS pore forming cargo effector; vasX	0.385393411
VC2674	ATP-dependent protease ATP-binding subunit; HslU	0.382376111
VC1585	catalase	0.378509766
VC0985	heat shock protein 90	0.372646378
VC1373	DnaK-related protein	0.368682857
VCA1060	3,4-dihydroxy-2-butanone 4-phosphate synthase ribB	0.362447606
VCA0386;VCA0312	parD3 and parD1 antitoxin hypothetical protein	0.362062904
VCA0116	T6SS recycling protease; clpV	0.361890930
VCA0291	site-specific recombinase IntI4	0.358570534
VCA0112	T6SS hypothetical protein; predicted membrane complex; vasC	0.356863976
VC0856	dnaJ protein	0.328421535
VC2197	flagellar hook protein; FlgE	0.326331303

VC0711	clpB protein	0.309919798
VC0276	bifunctional phosphoribosylaminoimidazolecarboxamide formyltransferase/IMP cyclohydrolase purH	0.307131082
VCA0197	guanosine 5'-monophosphate oxidoreductase	0.305691639
VCA0114	T6SS membrane complex protein; vasD	0.304550272
VCA0110	T6SS baseplate protein; vasA	0.294662915
VC0188	oligopeptidase A	0.285309334
VC0854	heat shock protein; GrpE	0.273338759
VC0424	hypothetical protein	0.261236140
VC0556	glutamate--cysteine ligase	0.259535497
VC0052	phosphoribosylaminoimidazole carboxylase catalytic subunit	0.258014281
VC0855	molecular chaperone DnaK	0.253726052
VC0342	iron-sulfur cluster-binding protein	0.232818107
VC0477	phosphoglycerate kinase pgk	0.232540797
VC2044	hypothetical protein	0.215146647
VC2389	carbamoyl phosphate synthase large subunit carB	0.209713917
VCA0005	hypothetical protein	0.208643919
VC1077	hypothetical protein	0.207402191
VC0275	phosphoribosylamine--glycine ligase	0.203069308
VCA0120	T6SS membrane complex protein; vasK	0.202494032
VC0051	phosphoribosylaminoimidazole carboxylase ATPase subunit	0.194501306
VC0543	recombinase A	0.193190268
VCA0637	dihydropteridine reductase	0.188420276
VC1483	3-hydroxydecanoyl-(acyl carrier protein) dehydratase	0.185845292
VC0186	glutathione reductase	0.177990329
VC0374	glucose-6-phosphate isomerase; pgi	0.177020508
VC2751	adenosine deaminase	0.176349793
VC2670	triosephosphate isomerase; tpiA	0.171415676
VC2394	preprotein translocase subunit; SecA	0.144433991
VC2775	tRNA uridine 5-carboxymethylaminomethyl modification enzyme; GidA	0.140049843
VC2764	F0F1 ATP synthase subunit beta	0.137417904
VC2720	putative DNA uptake protein	0.137019093
VCA0013	maltodextrin phosphorylase	0.133502567
glyA	serine hydroxymethyltransferase	0.130214809
ispH	4-hydroxy-3-methylbut-2-enyl diphosphate reductase	0.129959770
VC0394	excinuclease ABC subunit A ; uvrA	0.123902710

VC0521	O-sialoglycoprotein endopeptidase; gcp	0.117857361
VC0223	ADP-heptose--LPS heptosyltransferase II, putative	0.117303348
VC2766	F0F1 ATP synthase subunit alpha	0.112933807
VC2716	hypothetical protein	0.108732759
VC2660	elongation factor P	0.104389852
VC1000	acetyl-CoA carboxylase subunit beta	0.099589657
VC0695	phospho-2-dehydro-3-deoxyheptonate aldolase	0.098998883
VC2623	tryptophanyl-tRNA synthetase	0.098328265
VC0108	DNA polymerase I	0.096278320
VC0451	hypothetical protein	0.093923081
VC0190	DNA-dependent helicase II; uvrD	0.088613892
VC2765	F0F1 ATP synthase subunit gamma	0.080352199

[a]Proteins significantly enriched in Δ*lonA* relative to WT are shown. Student's t-test using a Benjamini-Hochberg FDR cutoff of 5% was used to identify proteins that were differentially expressed.
