# Supplementary material for: c-di-GMP inhibits LonA-dependent proteolysis of TfoY in Vibrio cholerae
Source: PLoS Genet. 2020 Jun 26;16(6):e1008897. doi: 10.1371/journal.pgen.1008897 (PMC7371385; doi:10.1371/journal.pgen.1008897)
Supplement: S2 Table — (PDF) [file pgen.1008897.s006.pdf]

Joshi et al.

S2 Table. Proteins enriched in WT relative to Δ*lonA*[a]

Gene	Description	Fold Change (log2)
VC1920	ATP-dependent protease LA; LonA	2.621082664
VC0620	peptide ABC transporter, periplasmic peptide-binding protein	0.77313396
VC1403	methyl-accepting chemotaxis protein	0.616571678
VC1523	hypothetical protein	0.588189086
VC1622	outer membrane protein, putative	0.580670401
VCA0697	sensory box/GGDEF family protein; cdgD	0.542207244
VC1621	agglutination protein	0.498378366
VC1441	cytochrome c oxidase, subunit CcoO	0.478708832
VC1424	spermidine/putrescine ABC transporter, periplasmic spermidine/putrescine-binding protein	0.409029135
VCA0849	hypothetical protein	0.400282145
VCA0405	hypothetical protein	0.397481483
VC1692	trimethylamine-N-oxide reductase	0.37527705
VCA0205	anaerobic C4-dicarboxylate transporter	0.365745926
VC2217	beta-N-acetylhexosaminidase	0.362460785
VC0490	hypothetical protein	0.342823848
VC1425	spermidine/putrescine ABC transporter, periplasmic spermidine/putrescine-binding protein	0.340334343
VC0538	thiosulfate ABC transporter, periplasmic thiosulfate-binding protein	0.300140437
VC0907	DL-methionine transporter ATP-binding subunit	0.291273999
VCA0807	ABC transporter, periplasmic substrate-binding protein	0.283885768
VC1844	cytochrome d ubiquinol oxidase, subunit I	0.266298023
VCA1043	tagE protein	0.260448366
VC2051	thiol:disulfide interchange protein DsbE	0.249364711
VC0156	vitamin B12 receptor	0.248729424
VC2259	sulfate adenylyltransferase subunit 1	0.22870666
VC2213	outer membrane protein OmpA	0.213343266

VCA0585	glutathione S-transferase, putative	0.212515607
VC1170	tryptophan synthase subunit beta	0.208670749
VC1680	peptide ABC transporter, periplasmic peptide-binding protein	0.201142681
VC0927	UDP-N-acetyl-D-mannosamine transferase	0.188408013
VC1414	thermostable carboxypeptidase 1	0.171369224
VCA0998	Oye family NADH-dependent flavin oxidoreductase	0.16395781
VC0918	UDP-N-acetyl-D-mannosamine dehydrogenase	0.145839381
VC1769	DNA methylase HsdM, putative	0.137020074
VC1021	LuxO repressor protein	0.121211053
VC1834	hypothetical protein	0.119616623
VC2510	aspartate carbamoyltransferase catalytic subunit	0.110111106
VC1503	hypothetical protein	0.109697749
VC1839	tolQ protein	0.089846289

[a]Proteins significantly enriched in WT relative to Δ*lonA* are shown. Student's t-test using a Benjamini-Hochberg FDR cutoff of 5% was used to identify proteins that were differentially expressed.
