# Supplementary material for: c-di-GMP inhibits LonA-dependent proteolysis of TfoY in Vibrio cholerae
Source: PLoS Genet. 2020 Jun 26;16(6):e1008897. doi: 10.1371/journal.pgen.1008897 (PMC7371385; doi:10.1371/journal.pgen.1008897)
Supplement: S3 Table — (PDF) [file pgen.1008897.s007.pdf]

Joshi et al.

S3 Table. COMSTAT2 analysis of WT, Δ*tfoY,* Δ*lonA,* and Δ*lonA* Δ*tfoY* biofilms grown for 24 hrs.

Strain	Biomass ($\mu m^3/\mu m^2$)	Mean Thickness (SD) (μm)		Substrate Coverage	Roughness Coeffecient
		Avg.	Maximum		
WT	12.21 (2.52)	12.46 (2.97)	16.45 (3.30)	0.76 (0.14)	0.15 (0.13)
Δ*tfoY*	12.08 (1.76)	12.61 (2.21)	17.12 (2.71)	0.82 (0.12)	0.12 (0.01)
Δ*lonA*	8.40 (4.40) *	11.26 (3.07)	21.29 (4.21) *	0.28 (0.12) ****	0.39 (0.19) ***
Δ*lonA* Δ*tfoY*	8.36 (2.74) *	11.34 (3.82)	20.72 (4.39) *	0.30 (0.10) ****	0.41 (0.11) **

a Quantitative analysis of biofilm formation by CLSM was performed using COMSTAT2. Statistcal analysis was performed with a One-way ANOVA with Tukey’s post-hoc analysis (*p<0.05, **p<0.01, ***p<0.001 ****p<.0001).
