# Supplementary material for: c-di-GMP inhibits LonA-dependent proteolysis of TfoY in Vibrio cholerae
Source: PLoS Genet. 2020 Jun 26;16(6):e1008897. doi: 10.1371/journal.pgen.1008897 (PMC7371385; doi:10.1371/journal.pgen.1008897)
Supplement: S4 Table — (PDF) [file pgen.1008897.s008.pdf]

S4 Table. Strains and plasmids used in this study.

FY#	*V. cholerae* Strains	Resistance	Reference
FY_1	*Vibrio cholerae* O1 El Tor, Smooth A1552 (Wild Type)	Rif	Yildiz FH, Dolganov NA, Schoolnik GK. 2001
FY_1053	Δ*ctxAB*	Rif	This study
FY_11013	Δ*ctxAB* Δ*lonA*	Rif	This study
FY_4507	Δ*lonA*	Rif	Rogers
FY_12974	*lonA*(S678A)	Rif	This study
FY_9789	Δ*lonA* Tn7::*lonA*	Rif, Gm	Rogers
FY_12953	Δ*tfoY*	Rif	This study
FY_7150	ΔcdgJ ΔrocS ΔlacZ (Δ2PDE)	Rif, Gm	This study
FY_6347	ΔcdgD ΔcdgH ΔcdgK ΔcdgL (Δ4DGC)	Rif, Gm	This study
FY_13650	Δ*tfoY* Tn7::*tfoY*	Rif, Gm	This study
FY_12956	ΔlonA Δ*tfoY*	Rif	This study
FY_13652	ΔlonA Δ*tfoY* Tn7::*tfoY*	Rif, Gm	This study
FY_13971	ΔlonA Δ*tfoY* Tn7::*lonA*	Rif, Gm	This study
FY_237	Wild Type Tn7::gfp	Rif, Gm	Lim B, Beyhan S, Meir J, Yildiz FH. 2006.
FY_13017	Δ*tfoY* Tn7::gfp	Rif, Gm	This study
FY_4512	Δ*lonA* Tn7::gfp	Rif, Gm	This study
FY_13016	ΔlonA Δ*tfoY* Tn7::gfp	Rif, Gm	This study
FY_14002	Wild Type Tn7::*ptac-tfoY*	Rif, Gm	This study
FY_14006	ΔlonA Tn7::*ptac-tfoY*	Rif, Gm	This study
FY_14208	Δ2PDE Tn7::*ptac-tfoY*	Rif, Gm	This study
FY_14209	Δ4DGC Tn7::*ptac-tfoY*	Rif, Gm	This study
FY_15961	ΔlonA Tn7::*ptac-tfoY*	Rif, Gm	This study
FY_15962	ΔlonA Δ2PDE Tn7::*ptac-tfoY*	Rif, Gm	This study
FY_15963	ΔlonA Δ4DGC Tn7::*ptac-tfoY*	Rif, Gm	This study
FY_15964	Δ*tfoY* Tn7::*ptac-tfoY*	Rif, Gm	This study
FY_15965	Δ*tfoY* ΔlonA Tn7::*ptac-tfoY*	Rif, Gm	This study
FY_15966	Δ*tfoY* Δ2PDE Tn7::*ptac-tfoY*	Rif, Gm	This study
FY_15967	Δ*tfoY* Δ4DGC Tn7::*ptac-tfoY*	Rif, Gm	This study
FY_15968	Δ*tfoY* Tn7::*ptac-tfoY* pBBR-*vipA-lux*	Rif, Gm, Cm	This study
FY_15969	Δ*tfoY* Δ2PDE Tn7::*ptac-tfoY* pBBR-*vipA-lux*	Rif, Gm, Cm	This study
FY_15970	Δ*tfoY* Δ4DGC Tn7::*ptac-tfoY* pBBR-*vipA-lux*	Rif, Gm, Cm	This study
FY_15971	Δ*tfoY* Tn7::*ptac-tfoY* pBBR-*hcp2-lux*	Rif, Gm, Cm	This study
FY_15972	Δ*tfoY* Δ2PDE Tn7::*ptac-tfoY* pBBR-*hcp2-lux*	Rif, Gm, Cm	This study

FY_15973	Δ*tfoY* Δ4DGC Tn7::*ptac-tfoY* pBBR-*hcp2-lux*	Rif, Gm, Cm	This study

FY#	Plasmid Harboring Strains	Resistance	Reference
pGP704SacB	pGP704 derivative; mob/oriT sacB	Amp	G. Schoolnik
pFY_5867	pGP704SacB-Δ*ctxAB*	Amp	This study
pFY_596	pGP704SacB-Δ*lonA*	Amp	Rogers et al. 2016
pFY_5532	pGP704SacB-Δ*tfoY*	Amp	This study
pFY_5523	pGP704SacB-*lonA* Full Length (For Site Directed Mutagenesis)	Amp	This study
pFY_5517	pGP704SacB-*lonA*(S678A)	Amp	This study
pFY_720	pGP704-Tn7	Amp, Gm	Lim B, Beyhan S, Meir J, Yildiz FH. 2006.
pUX-BF13	oriR6K helper plasmid, mobl oriT, provides the Tn7 transposition function in trans	Amp	Lim B, Beyhan S, Meir J, Yildiz FH. 2006.
pFY_117	pGP704-Tn7::*gfp*	Amp, Gm	Lim B, Beyhan S, Meir J, Yildiz FH. 2006.
pFY_3488	pGP704-Tn7::*lonA*	Amp, Gm	Rogers et al. 2016
pFY_5665	pGP704-Tn7::*tfoY* (Contains *tfoY* open reading frame and 500bp upstream)	Amp, Gm	This study
pFY_5833	pGP704-Tn7::*Ptac-tfoY*	Amp, Gm	This study
pFY_4431	pBBR-*hcp2-lux* (Contains 300bp upstream of VCA0017 open reading frame as well as 100bp into the gene)	Amp, Cm	This study
pFY_4434	pBBR-*vipA-lux* (Contains 400bp upstream of VCA0107 open reading frame as well as 100bp into the gene)	Amp, Cm	This study
